# Gut microbiome composition influences immunologic alterations in the blood and gut of HIV-positive and HIV-negative men who have sex with men

**DOI:** 10.3389/fimmu.2025.1707736

**Published:** 2026-01-02

**Authors:** Charles Preston Neff, Janet Siebert, Mallory Karr, Ricky Lippincott, Rachel Kvaal, Amy T. Noe, Elena Wall, Nichole Nusbacher, Suzanne Fiorillo, Blair P. Fennimore, Thomas B. Campbell, Catherine Lozupone, Brent E. Palmer

**Affiliations:** 1 Department of Medicine, University of Colorado Anschutz, Aurora, CO, United States; 2 Department of Biomedical Informatics, University of Colorado Anschutz, Aurora, CO, United States

**Keywords:** HIV, microbiome, men who have sex with men, colonic, inflammation, antiretroviral therapy, sexual behavior, CyTOF

## Abstract

**Background:**

HIV infection and factors associated with sexual activity among men who have sex with men (MSM) can dysregulate relationships between the gut microbiome and immune system.

**Methods:**

To explore these relationships in depth, blood and colonic biopsy samples from HIV+ and HIV- MSM and non-MSM were analyzed using Cytometry by Time of Flight (CyTOF). Immune profiles were then integrated with gut microbiome composition and MSM-related behaviors.

**Results:**

HIV infection status influenced immune cell composition in colonic biopsies, marked by a loss of CD4⁺ CD103⁺ and CD8⁺CD103⁺ tissue-resident T cells and group 3 innate lymphoid cells (ILC3s). In the blood, HIV status was linked to reductions in circulating group 2 innate lymphoid cells (ILC2s), and naïve CD8⁺ T cells, while mucosal-associated invariant T (MAIT) cells were reduced in MSM engaging in high-risk sexual behaviors regardless of HIV status. Network analysis revealed distinct, tissue-specific relationships between immune cell populations and gut microbial taxa, further shaped by both HIV infection and MSM-associated factors.

**Conclusions:**

These findings provide new insights into host:microbe interactions, with implications for immune regulation, HIV persistence, and transmission among MSM.

## Introduction

The intestinal mucosa harbors the body’s greatest number of activated CD4^+^ T cells expressing the HIV coreceptor CCR5 and is thus a primary location for HIV replication (1). Severe depletion of CD4^+^ T cells during HIV infection leads to immune dysfunction and breakdown of the intestinal barrier which allows for increased bacterial translocation (2). These processes are tightly correlated with HIV disease progression and development of comorbidities, such as metabolic syndrome (3), that are prevalent in people with HIV (PWH). However, not all immune alterations associated with HIV infection are directly related to CD4^+^ T cell depletion. For example, colon biopsies from PWH demonstrate increased frequencies of activated CD8^+^ T cells and Th17 cell loss, which is associated with mucosal barrier disruption and microbial translocation (4, 5). Additional work has shown expansion of pro-inflammatory myeloid cells and altered regulatory T cell frequencies in the colonic mucosa (6). More recent studies highlight skewing of innate lymphoid cells, particularly group 3 innate lymphoid cells (ILC3s), with decreased IL-22–producing subsets (7). Prior studies of PWH (8, 9) have typically examined only a few targeted immune populations in the gut due to limited cells obtained by biopsy. However, newer approaches for simultaneous examination of more immune markers, such as time-of-flight mass cytometry (CyTOF), now enable deeper characterization of the immune system even from cell-limited colonic biopsies. CyTOF has been particularly useful in characterizing immune cells from the gut, as exemplified in other disease contexts, such as celiac and Crohn’s disease (10).

Men who have Sex with Men (MSM) make up the majority of PWH in the United States and Europe. Thus most studies have HIV^+^ cohorts dominated by MSM, yet few of these studies include HIV seronegative MSM controls engaging in sexual behaviors that promote HIV transmission. Recent studies of HIV seronegative MSM (HIV^-^ MSM) have identified distinct immune profiles (11–13), some of which had been previously attributed to HIV infection. Furthermore, we and others have shown that in Western countries, the gut microbiome in HIV^-^ and HIV^+^ MSM is different from men who have sex with women (MSW) and women, resembling the Prevotella-rich/Bacteroides poor microbiomes of non-Western cultures (14, 15). These microbiome differences in MSM have been linked with sexual behaviors including having >3 recent sexual partners and lack of condom use (14). Although some studies have linked these changes to receptive anal intercourse (RAI) (16) and hyperosmolar lubricants (17), studies from our group and others have found no association with RAI (14, 15, 18). The effects of this microbiome-type on the gut immune system are also not well understood. Interestingly, fecal bacteria from MSM have been shown to drive immune activation (13, 19) and elevate the expression of the HIV coreceptor CCR5 on CD4^+^ T cells (20), both of which can promote HIV transmission during anal intercourse (12, 13, 15, 21–25). Accordingly, an altered gut microbiome has been proposed as a risk-factor for transmission among MSM (26) and gut microbiome signatures among MSM also differentiated HIV seroconverters from uninfected individuals (27). HIV-infection has also been associated with distinct differences in microbiome composition, such as increases in Proteobacteria and decreased diversity, including loss of commensals that can produce beneficial immune-modulatory metabolites such as butryate (28). HIV-associated immune dysfunction can also impact host:microbe interactions (29), in particular because CD4^+^ T cells play a critical role in the control of commensal and pathobiont species in the gut (30). While successful antiretroviral therapy (ART) partially reverses immune dysregulation and HIV-associated alterations in microbiome composition, neither are fully normalized (31, 32). Furthermore, disruption in relationships between gut microbes and the host is proposed to be a driver of chronic inflammation impacting PWH on ART (9).

Studies of PWH have often used blood and fecal samples for immune and microbiome profiling respectively (8, 33–35), rather than more difficult to obtain colonic mucosal samples that can reveal host and microbiome relationships where they closely interface (8, 9, 36, 37). To comprehensively analyze the effects of HIV infection and the MSM microbiome on the immune repertoire in PWH, here we perform deep immune phenotyping of colonic biopsy and blood using CyTOF to examine the relationship between immune cell populations and microbiome composition of colonic biopsy and matched feces using 16S ribosomal RNA (rRNA) sequencing. We hypothesized that HIV-associated immune-dysfunction and the MSM-associated microbiome would alter the relationship between intestinal microbes and immune populations, and that this effect would be most pronounced in biopsy. Furthermore, to disentangle the effects of the MSM-associated microbiome/behaviors and HIV infection on the immune system we examined a control cohort of HIV- MSM in addition to ART treated MSM with HIV (HIV^+^ MSM) and HIV seronegative non-MSM controls (HIV^-^ non-MSM). Using this approach, we found that several immune populations, such as Mucosal-Associated Invariant T (MAIT) cells and naïve T cells, that were previously thought to be altered by HIV, were also altered in HIV^-^ MSM. We also identified HIV associated differences, such as the loss of colonic ILC3s and CD4^+^CD103^+^ and CD8^+^CD103^+^ T cells. In addition, we found that various aspects of MSM behavior were associated with immune activation and the presence of pathobionts in the gut. Lastly, network analysis revealed associations between immune populations and gut microbes that were influenced by HIV infection or MSM status. Taken together, these data highlight new host:microbe interactions and propose that HIV-associated immune dysfunction and particular behaviors among MSM that can influence them.

## Results

### Study participant demographics

Three cohorts of individuals were studied: 1) 33 ART treated MSM living with HIV (HIV^+^ MSM), 2) 16 HIV seronegative MSM (HIV^-^ MSM) and 3) 21 HIV seronegative non-MSM (control; HIV^-^ non-MSM), including 11 men who have sex with women (MSW) and 10 women (HIV-F). HIV seronegative MSW and women were combined in the control population, and gender, age and body mass index (BMI) were controlled for statistically. Visit age was statistically different between HIV^-^F and HIV^-^MSW and between HIV^-^non-MSM and HIV^+^MSM. BMI was statistically different between HIV^-^F and HIV^-^MSW. Demographics of the study population are given in **Table 1** and further details on exclusion/inclusion are in the Methods. All study participants were recruited from the greater Denver Metropolitan area, Colorado, USA.

**Table 1:** Demographics of the study population.

Variables	HIV^-^ non-MSM		HIV^-^ MSM	HIV^+^ MSM
	HIV^-^ F	HIV^-^ MSW		
N	10	11	16	33
Race (C/AA/O)	10/0/0	7/1/3	13/1/2	29/4/0
Hispanic (yes/no)	3/7	6/9	5/11	4/29
Visit Age (yrs)	33.4 (5.0)	40.2 (12.5)^#^	42.5 (11.0)	48.4 (10.5)***
BMI	23.1 (3.3)	27.1 (2.4)^#^	26.4 (2.3)	25.3 (3.0)
Bristol Stool Scale	3.7 (1.4)	3.9 (1.5)	4.1 (1.6)	4.4 (1.4)
CD4 T cell count (cells/uL)	NA	NA	NA	727 (345)
Plasma HIV RNA Viral Load (copies/mL)	NA	NA	NA	5.73 (11.61)

Significant differences across groups are shown for continuous variables. Data is shown as mean with standard deviation. C, Caucasian; AA, African American; BMI, Body Mass Index; F, female; MSW, Men who have sex with women; MSM, Men who have sex with men; #, p<0.05 HIV^-^F vs. HIV^-^MSM; *, p<0.05; ***, p<0.001 HIV^-^non-MSM vs. HIV^+^MSM; ANOVA with Tukey HSD to correct for multiple comparisons.

### Immune cell differences with MSM and ART-treated HIV infection in blood and gut biopsy

**Figure 1:**
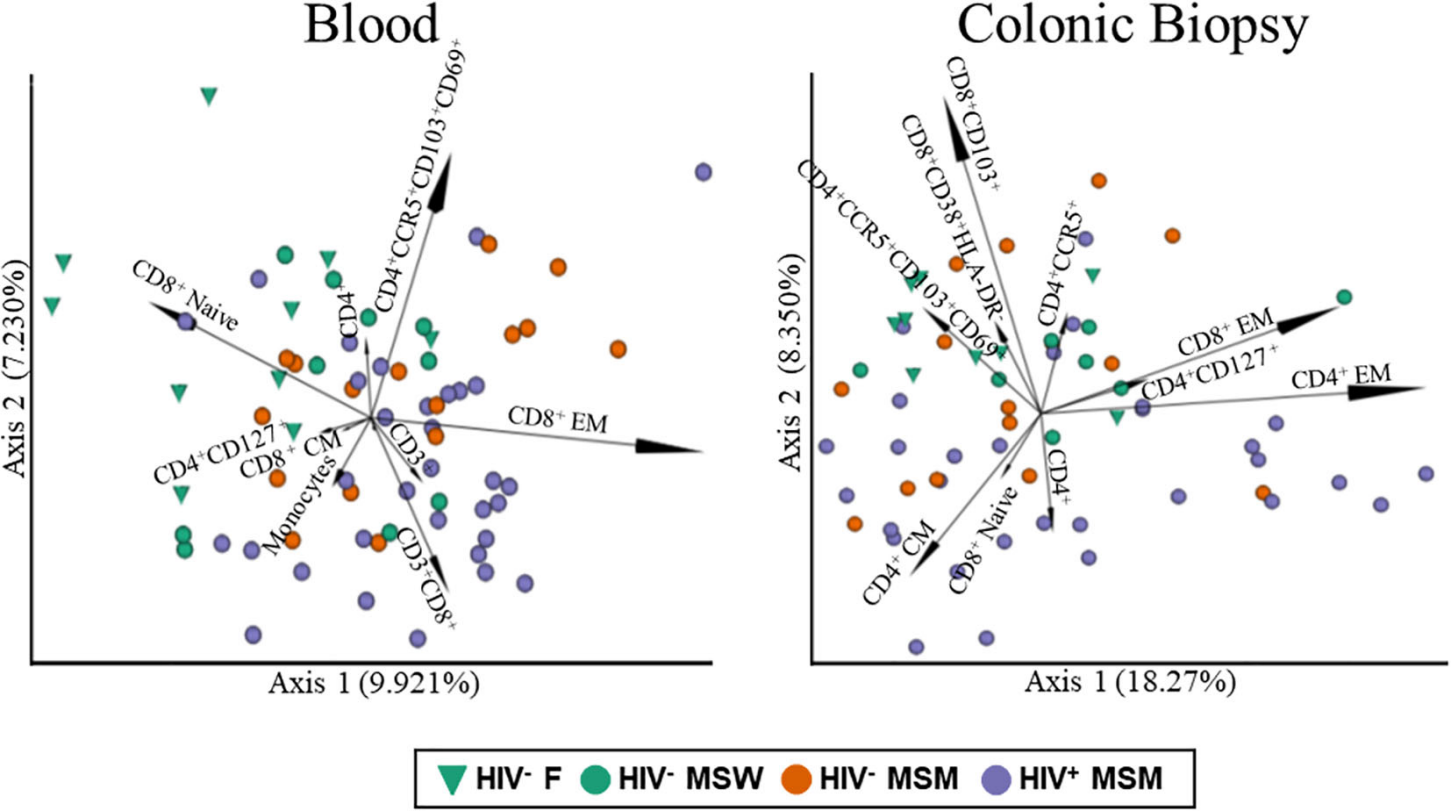
PCoA plots of blood and colonic biopsy immune cell populations. PCoA plots were made using Canberra distance matrices of immune data from blood (left panel) and colonic biopsy (right panel). Points are colored by cohort: Green triangles = HIV^-^ females, Green circles = HIV^-^ MSW, Orange circles = HIV^-^ MSM and Purple circles = HIV^+^ MSM. Vector magnitude of immune populations is related to importance, calculated across all dimensions using the biplot function of QIIME 2.

To characterize immune cell differences in the colon and blood associated with MSM status and ART-treated HIV infection, we employed CyTOF with an extensive monoclonal antibody panel (mAb) (**Supplementary Table 1**). The mAb panel targeted a wide variety of immune populations with a focus on CD4+ and CD8+ T cells, including markers for T regulatory cells (Tregs), Th17, Th1, Tfh, T cell maturation, inhibitory receptors, gut homing receptors, markers of acute/chronic activation, and Mucosal Invariant T Cells (MAITs). The panel also identified populations of monocytes, macrophages, dendritic cells, B cells, Natural Killer (NK) cells, and NK T cells for a total of 49 populations in the blood and 48 in the biopsy. Participant blinded analysis focused on these immune populations because they had distinct or bimodal staining patterns which ensured accurate and reproducible gating. The representative gating strategy is shown in **Supplementary Figure 1**. To assess how immune cell populations varied across individuals and cohorts, pairwise distances were calculated using the Canberra metric and visualized via Principal Coordinates Analysis (PCoA, **Figure 1**). The effects of HIV infection, MSM status and confounders were determined with the adonis test. Since samples were run fresh and thus at different times, we also included time of sample collection (date) as a potential confounding variable by running the models d ~ age + sample_collection_time + MSM_status + HIV_status + gender. In colonic biopsies, immune cell composition significantly differed by HIV infection status (p = 0.004), but not by MSM status (p = 0.158). Sample collection date (p = 0.001), but not sex (p = 0.103) or age (p = 0.172), also influenced clustering. In blood, immune populations similarly clustered by HIV infection (p = 0.008), while sex (p = 0.002), sample collection date (p = 0.001), and age (p = 0.001) were significant contributors to variation. All downstream analyses were therefore adjusted for age, sample collection date, and sex to account for potential confounding effects.

To better understand how specific immune cell populations varied by HIV and MSM status, we plotted the frequency of each immune population and performed statistical comparisons between compartments and cohorts. Immune populations that significantly differed between blood and colon are shown on the left side of **Supplementary Figure 2** and detailed in **Supplementary Table 2**. T cell populations elevated in blood relative to colon included CD3^+^, CD3^+^CD4^-^CD8^-^, CD8^-^ MAIT, CD4^+^CD25^+^, CD4^+^ central memory (CM), CD4^+^ naïve, CD4^+^CD38^+^HLA-DR^-^, CD4^+^CD127^+^, CD8^+^, CD8^+^ effector memory terminally differentiated (EMTD), CD8^+^ naïve, CD8^+^CD127^+^, and CD8^+^ MAIT cells. In contrast, T cell populations enriched in the colon included CD4^+^ effector memory (EM), CD4^+^CD69^+^, CD4^+^CD90^+^, CD4^+^CD103^+^, CD4^+^CCR5^+^, CD4^+^PD-1^+^, T follicular helper (Tfh), CD4^+^CD103^+^CD69^+^, CD4^+^CD103^+^CD69^+^CCR5^+^, CD8^+^ EM, CD8^+^CD38^+^HLA-DR^-^, CD8^+^CD69^+^, CD8^+^CD103^+^, and CD8^+^PD-1^+^ cells. These findings are consistent with what is known about T cells blood and tissue (38) with increases in naïve populations in the blood not commonly found in tissue and increases in tissue homing markers (CD103 (39)), activation/exhaustion markers (CD69 (39, 40) and PD-1 (41)) and effector T cells in the colon (42), all of which tend to be expressed at low levels in transiting T cells in the blood (43).

**Figure 2:**
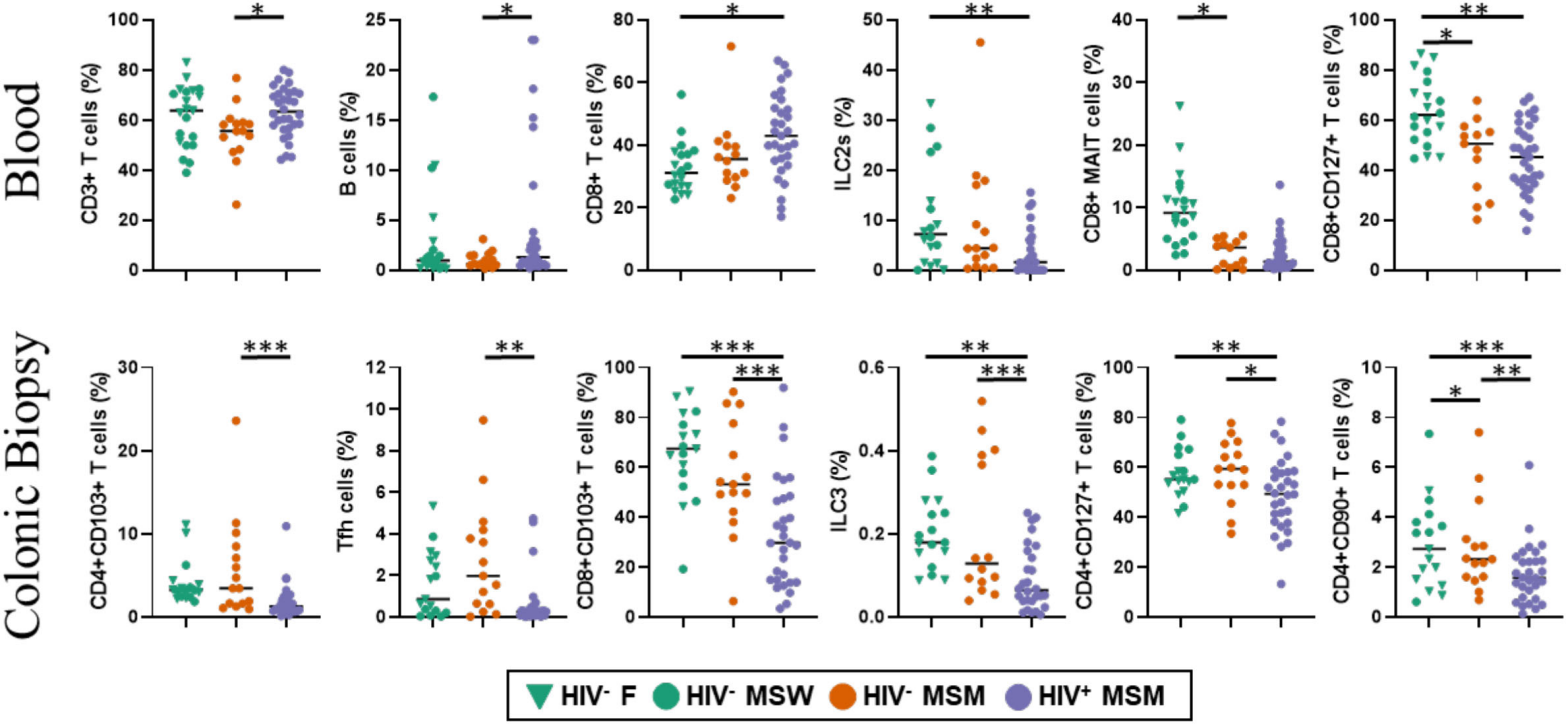
Immune populations that differ significantly across cohorts. Dot plots display the distribution of immune cell populations identified as statistically significantly different between cohorts, as determined in **Supplementary Figure 2**. The top row represents immune populations from blood; the bottom row represents those from colonic biopsies. Green triangles represent HIV^-^ females, green circles represent HIV^-^ MSW (men who have sex with women), orange circles represent HIV^-^ MSM (men who have sex with men), and purple circles represent HIV^+^ MSM. For simplicity, HIV^-^ females and HIV^-^ MSW are grouped as HIV^-^ non-MSM in analyses. We computed pairwise significant differences across cohorts using a linear model of the form analyte ~ age + day + gender + cohort, with Tukey’s HSD correction for multiple comparisons. Significance codes are defined as ‘***’ [0, 0.001], ‘**’ (0.001, 0.01], ‘*’ (0.01, 0.05); with square brackets indicating endpoints included in the interval.

Six immune populations in blood and six in colon were significantly different across cohorts (right side of **Supplementary Figure 2**). Some differed by HIV status and some by MSM status. To further explore these findings, we plotted the frequencies of each significant population by cohort (**Figure 2**). In blood, CD3^+^ T cell frequencies were significantly higher in HIV^+^ MSM compared to HIV^-^ MSM, suggesting a depletion of total T cells associated with MSM status. As expected, CD4^+^ T cell frequencies in both blood and colon were reduced in HIV^+^ MSM compared to the other cohorts (**Supplementary Figure 2**), though this did not reach statistical significance. Blood CD8^+^ T cells were elevated in HIV^+^ MSM compared to both HIV^-^ MSM and HIV^-^ non-MSM, reaching statistical significance only relative to HIV^-^ non-MSM (p = 0.013), consistent with cytotoxic T cell expansion in chronic HIV infection and likely related to the increase in total CD3^+^ T cells in HIV+ MSM. Surprisingly, B cells were increased in HIV^+^ MSM compared to HIV^-^ non-MSM. Since CyTOF does not include a lymphocyte-specific (size vs granularity discriminator) gate, this reduction may reflect an increase in other CD45^+^ immune populations not assessed here.

Group 2 Innate lymphoid cells (ILC2) were lower in blood from HIV^+^ MSM individuals, reaching significance when compared to HIV^-^ non-MSM (p = 0.004). Interestingly, we also identified changes in immune population frequencies that were driven by MSM status. For example, blood CD8^+^ MAIT cells were decreased in HIV^-^ MSM compared to HIV^-^ non-MSM (p = 0.01), suggesting an MSM-associated loss of these cells which are known to be modulated by bacterial metabolites (44). Blood CD8^+^CD127^+^ T cells, likely representing naïve or early memory cells, were significantly reduced in both HIV^-^ MSM (p = 0.021) and HIV^+^ MSM (p = 0.002) relative to HIV^-^ non-MSM, indicating a combined effect of MSM status and HIV on these cells.

In the colonic biopsies, CD4^+^CD103^+^ and CD8^+^CD103^+^ T cells, markers of mucosal residency, were significantly reduced in HIV^+^ MSM compared to HIV^-^ MSM (p = 0.0002 and p = 0.004, respectively), and CD8^+^CD103^+^ cells were also significantly lower than in HIV^-^ non-MSM (p = 0.001). These findings suggest that chronic HIV infection disrupts gut-resident memory T cells, potentially contributing to epithelial barrier dysfunction and microbial translocation. Notably, no differences in these populations were observed in peripheral blood, underscoring the importance of mucosal tissue sampling in HIV studies. The frequency of colonic CD4^+^PD-1^+^CXCR5^+^ Tfh cells was also lower in HIV^+^ MSM compared to HIV^-^ MSM but did not reach statistical significance (p = 0.051), consistent with disruption of mucosal niches critical for B cell help and germinal center formation. Tfh cells were nearly absent in blood across all cohorts, reflecting their tissue-restricted nature.

Colonic ILC3s (45), defined as CD45^+^Lin^-^CD127^+^CD161^+^CD90^+^ICOS^-^ were significantly depleted in the HIV^+^ MSM cohort compared to both HIV^-^ non-MSM (p = 0.005) and HIV^-^ MSM (p = 0.0004), supporting the previously reported loss of ILC3s during HIV infection (46). ILC3s are critical for IL-22 production and epithelial barrier maintenance (45). Finally, CD4^+^CD127^+^ T cells in the colon, typically naïve cells, were decreased in HIV^+^ MSM, consistent with HIV-associated naïve T cell depletion. CD4^+^CD90^+^ T cells, which may represent effector subsets, were highest in HIV^-^ non-MSM, followed by HIV^-^ MSM, and lowest in HIV^+^ MSM, suggesting additive effects of MSM status and HIV on this population.

### Sexual behaviors associate with alterations in immune cell populations in MSM

To assess the impact of sexual behaviors on immune parameters in MSM, immune cell frequencies were analyzed in relation to behavioral data obtained via questionnaire. Engaging in receptive anal intercourse (RAI) was associated with significantly elevated levels of activated T cells, including blood CD8^+^CD38^+^HLA-DR^+^ and colonic CD4^+^CD38^+^HLA-DR^+^ T cells (**Supplementary Figure 3A**). Additionally, participants with three or more sexual partners within six months of their study visit date exhibited reduced frequencies of blood CD4^+^ central memory (CM) T cells (**Supplementary Figure 3B**). Lubricant use was associated with increased frequencies of colonic CD4^+^, NKT, and CD4^+^PD-1^+^ T cells (**Supplementary Figure 3C**), while individuals who had experienced a recent sexually transmitted infection (STI) displayed higher levels of naïve CD4^+^ T cells in the blood compared to those without a recent STI (**Supplementary Figure 3D**).

### Microbiome differences across MSM and ART-treated PWH in feces and colonic biopsy

**Figure 3:**
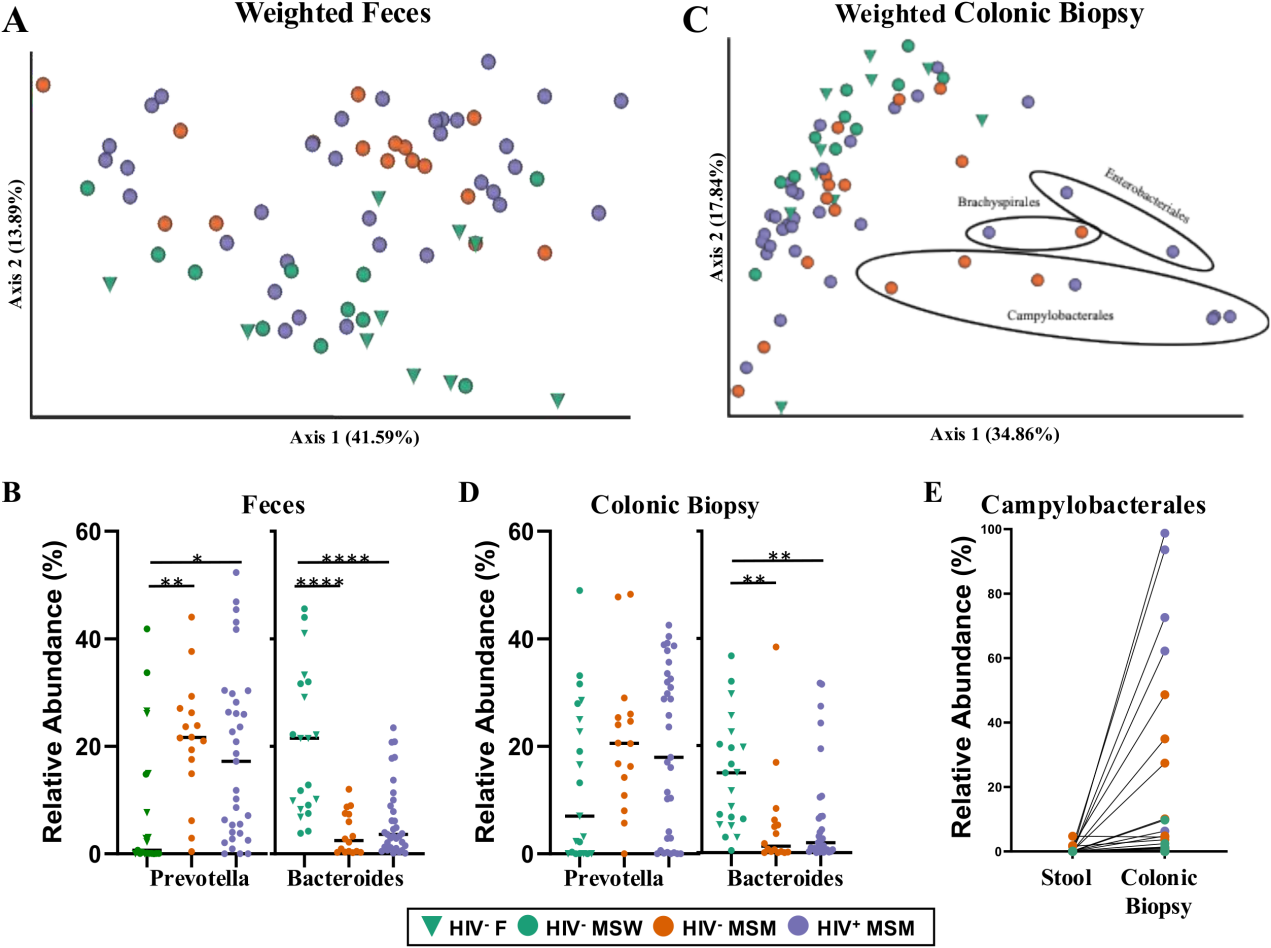
Microbiome composition of feces and colonic biopsy samples. **(A)** PCoA of Weighted UniFrac of feces colored by cohort. **(B)** Relative abundance of the genus Prevotella and genus Bacteroides in each of the 3 cohorts. **(C)** Same as A but for colonic biopsy samples. **(D)** Same as B but for colonic biopsy samples. **(E)** The relative abundance of bacteria in the Campylobacterales order detected in colonic biopsy versus matched fecal samples. Points colored by cohort. Statistical significance for Prevotella versus Bacteroides was evaluated with Kruskal-Wallis and Dunn’s *post hoc* test. Significance codes are defined as ‘***’ [0, 0.001], ‘**’ (0.001, 0.01], ‘*’ (0.01, 0.05); with square brackets indicating endpoints included in the interval.

Microbiome composition was evaluated in colonic biopsy samples and matched fecal specimens using 16S rRNA gene sequencing. The microbiome composition of the biopsies was significantly different from that of feces with both weighted (adonis: p=0.006) and unweighted (adonis p=0.001) UniFrac (47, 48). PCoA analysis of weighted (**Figure 3**) and unweighted UniFrac (**Supplementary Figure 4**) distances showed that a subset of colonic biopsy samples clustered closely with fecal samples and a subset had highly differentiated microbiomes.

When considering only feces, samples clustered primarily by MSM and not HIV-infection status with both weighted (**Figure 3A**) and unweighted UniFrac (**Supplementary Figure 4**), as has been observed previously (15, 25). Adonis showed a significant difference with MSM status (unweighted p=0.002, weighted p=0.001) and a trend toward significance with HIV status (unweighted p =0.06; adonis with formula: distance matrix ~ MSM-status + HIV-status). As previously reported (15, 25), both HIV^+^ and HIV^-^ MSM had a higher relative abundance of the genus *Prevotella* and lower *Bacteroides* compared to non-MSM in feces (**Figure 3B**; Kruskal Wallis with Dunn’s *post hoc*). Although MSM status had a significant effect on microbiome composition in colonic biopsy (unweighted UniFrac p=0.008 adonis; **Supplementary Figure 4**, weighted UniFrac p=0.031 adonis **Figure 3C**), the effect size was smaller compared to in feces (unweighted R^2^ = 0.071 biopsy versus 0.094 in feces; weighted R^2^ = 0.089 in biopsy versus 0.13 in feces). The microbiome composition in colonic biopsy was not significantly different with HIV-infection status with weighted or unweighted UniFrac (adonis with model distance matrix ~ MSM-status + HIV-status); Although *Bacteroides* was lower in relative abundance in colonic biopsy of HIV^+^ and HIV^-^ MSM compared to HIV^-^ non-MSM, *Prevotella* was not significantly different across cohorts in colonic biopsy (**Figure 3D**). With weighted UniFrac of biopsy, a subset of HIV^+^ and HIV^-^ MSM separated from the other biopsy samples across PC1 (**Figure 3C**). These samples were each dominated by a single microbial taxon often highly related to pathogens that can spread by fecal-oral transfer including *Brachyspirales*, *Enterobacteriale*s, or most commonly *Campylobacterales* (*Campylobacter* or *Helicobacter/Flexispira*) (**Figure 3C**). *Campylobacterales* made up 50-99% of the bacteria on 4 biopsies of HIV^+^ MSM samples and 24-50% in 3 HIV^-^ MSM even though *Campylobacterales* were only <5% of the bacteria observed in their feces (**Figure 3E**).

To identify Amplicon Sequence Variants (ASVs; unique sequences representing a single microbial taxon defined using DADA2 (49)) that differed across cohorts, we modeled relative abundance as a function of the number of reads (to correct for differences in sampling depth) and cohort. Since use of colonic biopsy samples has been proposed to be superior for detecting disease-associated changes in immune-modulatory bacteria that adhere closely to the gastrointestinal mucosal epithelium (36, 50, 51), we compared observed differences between cohorts across feces and colonic biopsy. **Supplementary Table 3** provides a summary of ASVs that had an adjusted p-value <0.1 for either feces or colonic biopsy. Fecal samples had a higher number of differing ASVs (18 in feces versus 8 in biopsy) and only 4 were significant in both. HIV^-^ MSM had 13 ASVs that differed from HIV^-^ non-MSM controls including increases in ASVs assigned as *Prevotella stercorea* and *Prevotella copri* and decreases in 11 ASVs that included *Bacteroides uniformis*, *Alistipes putredinis*, and diverse Firmicutes including *Faecalibacterium prausnitzii* and *Ruminococus bromii.* HIV infection was associated with a reduction in the relative abundance of 5 ASVs in feces, of which 4 were in the *Lachnospiraceae/Ruminococcaceae* families. These results are largely in line with what has previously been observed to differ in MSM and HIV in prior studies (28). Surprisingly, fewer differences were observed across cohorts in biopsy, and these were ASVs that were decreased in HIV^+^ and HIV^-^ MSM cohorts compared to the HIV^-^ non-MSM controls, with no HIV associated differences detected.

### Sexual behaviors associate with gut microbial composition in MSM

To assess the impact of sexual behaviors on microbiome parameters in MSM, relative abundance of bacteria were analyzed in relation to behavioral data obtained via questionnaire. Behavioral variables also corresponded with shifts in gut microbial composition. MSM engaging in RAI had decreased relative abundance of *Marseillibacter massiliensis* and *Coprococcus catus* in the colonic microbiome, as well as reduced levels of *Clostridium disporicum* in fecal samples (**Supplementary Figure 5A**). Those reporting three or more sexual partners over the last 6 months since study period showed lower abundance of *Odoribacter* sp*lanchnicus* in the colonic microbiome and *C. catus* in feces, along with increased abundance of *Peptoniphilus harei* in colonic biopsies (**Supplementary Figure 5B**). These findings underscore the influence of specific sexual behaviors on gut microbial ecology in MSM, independent of HIV status, and highlight behavioral factors as important modulators of the gut–immune interface.

### Integrative analysis of immune cell populations and microbiome

**Figure 4:**
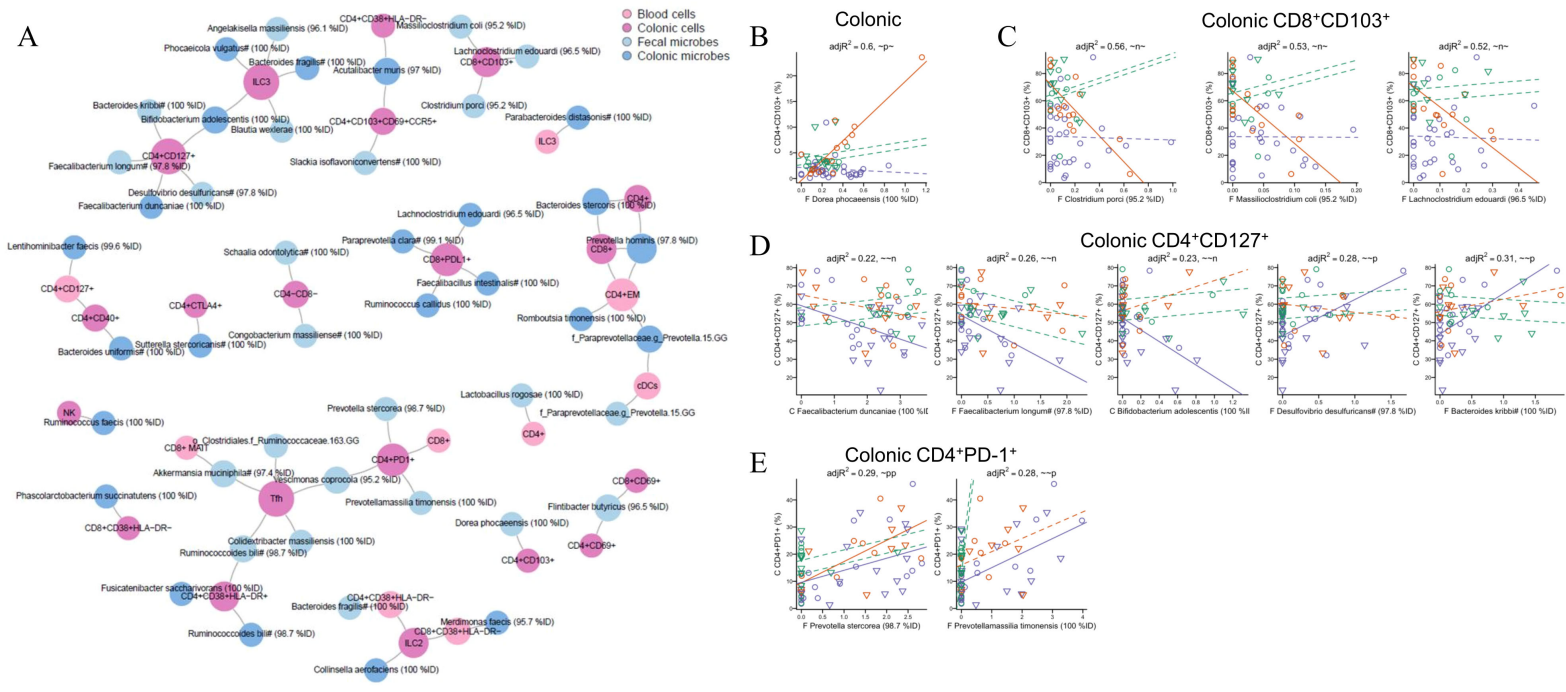
Integrated network of immune cell:microbe relationships. **(A)** Data from 4 sample types (blood cells, colonic cells, fecal microbes, and colonic microbes) support pairwise regression models across 5 assay pairs with one slope per cohort. Included relationships have an FDR-adjusted p-value <0.05 and at least one of the slopes being different than 0 with p < 0.005. Each node represents an analyte and each edge a relationship. The size of each node is a function of the number of its immediate neighbors. Nodes are color-coded by sample type. The network consists of 51 nodes and 46 edges. B = blood, F = fecal, C = colonic. **(B-E)** Detailed plots of fitted regression models for selected edges. Each circle represents observed data from one participant. Line colors correspond to cohorts with solid lines representing estimated slopes significantly different than 0 and dotted lines representing estimated slopes not significantly different. The adjusted R^2^ value and slope pattern is provided for each plot. The slope pattern consists of one term for each cohort (HIV^-^ non-MSM, HIV^-^ MSM, and HIV^+^ MSM), with p and n representing positive and negative slopes significantly different than 0, and ~ not significantly different. Microbes are reported as arcsinh-transformed relative abundance.

To identify relationships between immune cell populations and microbial ASVs, pairwise linear regressions were performed. Linear regressions were performed across 5 assay pairs: blood cells:colonic cells, blood cells:colonic microbes, blood cells:fecal microbes, colonic cells:colonic microbes, colonic cells:fecal microbes. The regressions included confounders and had an interaction term allowing immune:microbe relationships to vary by cohort. The resulting network was visualized using VOLARE (52) (**Figure 4A**). All network edges are described in **Supplementary Table 4**. The network was dominated by relationships between biopsy immune populations and microbial ASVs in biopsy (n=22) or feces (n=23), underscoring the localized nature of immune-microbiome interactions. In contrast, fewer associations were observed between blood immune populations and either colonic (n=7) or fecal microbes (n = 4), and between blood and colonic immune compartments (n=5). Immune cell populations that most frequently associated with microbial taxa included mucosal CD4^+^CD127^+^ naïve, Tfh, CD4^+^CD38^+^HLA-DR^+^, CD4^+^PD-1^+^, CD8^+^CD103^+^, CD8^+^, and CD8^+^PD-1^+^ T cells and ILC3s.

**Figure 5:**
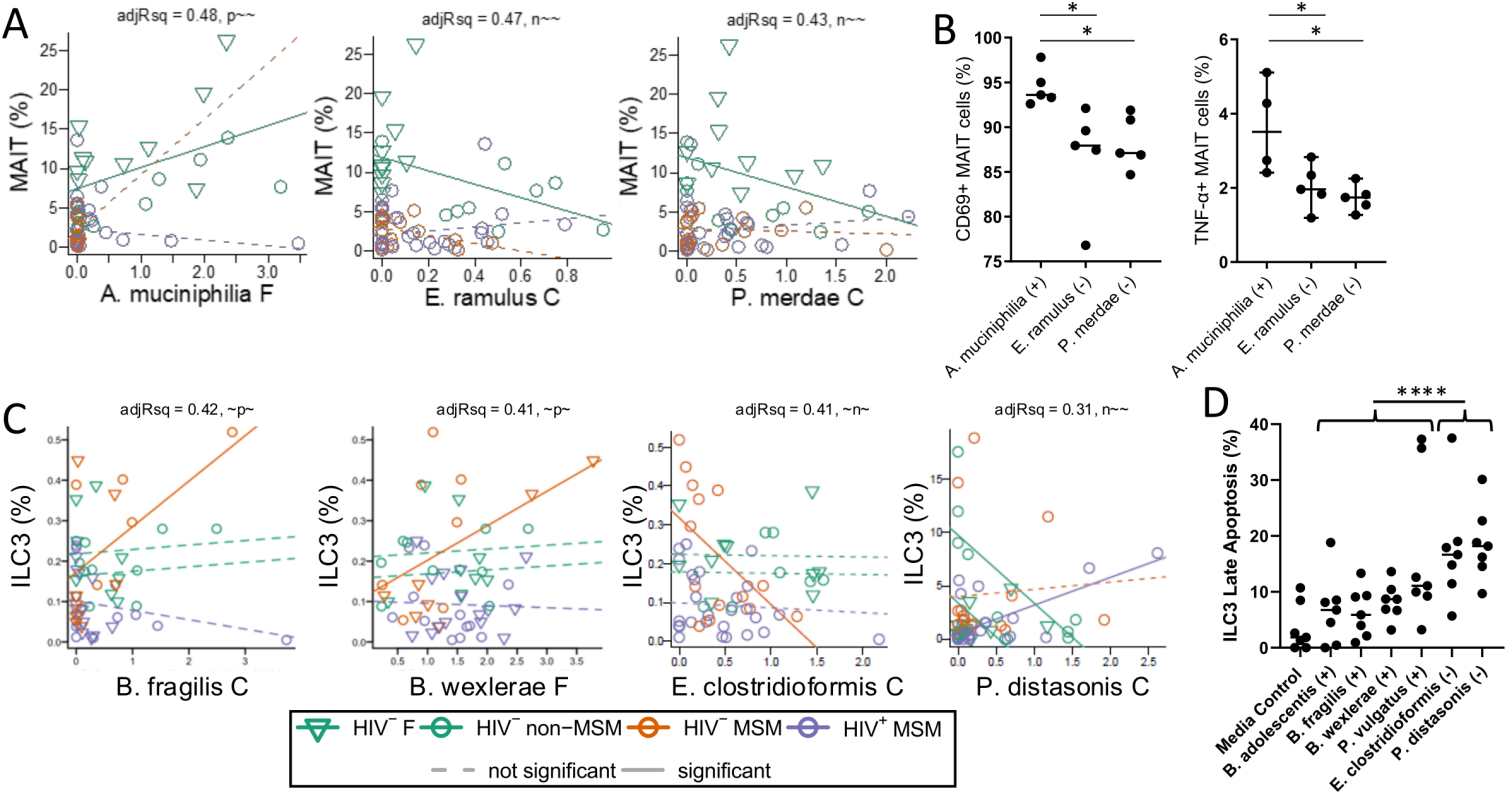
Bacteria associated with MAIT and ILC3 cell frequency *in vivo* induce activation and apoptosis when cultured *in vitro*. **(A)** Detailed plots of fitted regression models for blood MAIT cells as a function of *A*. *muciniphila* that positively associated with MAIT cells and *E. ramulus* and *P. merdae* that negatively associated with MAIT cells. F = fecal, C = colonic. Each circle represents observed data from one participant. Line colors correspond to cohorts with solid lines representing estimated slopes significantly different than 0 and dotted lines representing estimated slopes not significantly different than 0. The adjusted R^2^ value and slope pattern is provided for each plot. The slope pattern consists of one term for each cohort (HIV^-^ non-MSM, HIV^-^ MSM, and HIV^+^ MSM), with p representing significantly different than 0 (slope p < 0.05) and positive, n representing significantly different and negative, and ~ not significantly different (e.g. ~~n) **(B)** (Left) The frequency of MAIT cells expressing the CD69 activation marker after a 24 hour culture with fixed bacteria. Horizontal bars represent group medians. (Right) The frequency of MAIT cells expressing TNF-α after 6 hour culture with bacterial supernatants. **(C)** Detailed plots of fitted regression models for colonic ILC3s as a function of *B*. *fragilis and B. wexlerae* that positively associated with ILC3s and *E*. *clostridioformis* and *P. distasosnis* that negatively associated with ILC3s. **(D)** Flow cytometry analysis showing the percentage of apoptotic (PI^+^Annexin-V^+^) colonic ILC3s after 20 h culture with fixed bacteria that were positively (+) negatively (-) associated with ILC3 frequency. Statistical significance was calculated using Kruskal-Wallis. Significance codes are defined as ‘***’ [0, 0.001], ‘**’ (0.001, 0.01], ‘*’ (0.01, 0.05); with square brackets indicating endpoints included in the interval.

Among the most striking findings were five ILC3-microbe correlations, all of which were present only in HIV^-^ MSM and not in HIV^+^ MSM, suggesting relationships that are potentially disrupted by HIV infection (**Figure 4**). Positive associations were with *Bacteroides fragilis*, *Bifidobacterium adolescentis*, *Phocaeicola* (formally Bacteroides) *vulgatus*, and *Blautia wexlerae*. The only significantly negative association was with a poorly defined ASV whose closest relative was *Angelakisella massiliensis* (96.1%ID) (**Figure 5**). *B. fragilis* has been shown to promote colonic mucosa regeneration in colitis via promotion of IL-22 secretion by ILC3s (53), but Bacteroides sphingolipids have also been shown to exacerbate colitis by inhibiting ILC3-derived IL-22 production (54). P*. vulgatus* has also been shown to decrease IL-22 production by ILC3s, potentially though its influence on bile acid pools (55). *B. adolescentis* has also been reported to inhibit IL-22 production (56), suggesting these microbial interactions may variably support or impair ILC3 function depending on species, strain, or context. All correlations between ASVs and CD4^+^ and CD8^+^ T cells expressing the CD103 marker of mucosal residency were also present only in HIV^-^ MSM and not in HIV^+^ MSM (**Figures 4B, C**), suggesting relationships that are potentially disrupted by HIV infection, consistent with these cell populations being significantly depleted in the mucosa of HIV^+^ MSM.

In contrast, CD4^+^CD127^+^ T cells were associated with five ASVs, but only within the HIV^+^ MSM group, suggesting HIV infection enhances microbe associations with this immune population (**Figure 4D**). CD127, the alpha chain of the IL-7 receptor, is expressed on naïve and memory T cells and is often reduced during HIV infection. Its expression has been linked to immune recovery and naïve T cell preservation (57). In HIV^+^ MSM *Faecalibacterium duncaniae* (100% ID), *Faecalibacterium longum* (97.8% ID), and *B. adolescentis* (100%ID) were negatively associated with CD4^+^CD127^+^ T cell frequencies. Since these are generally considered beneficial/anti-inflammatory bacteria, a negative correlation with naïve CD4^+^ T cells suggests a dysregulated relationship with HIV. Conversely, *Desulfovibrio desulfuricans* and *Bacteroides kribbi* showed positive correlations with CD4^+^CD127^+^ T cells. *D. desulfuricans*, has been variably associated with inflammation and dysbiosis (58) but specific research focusing on *B. kribbi* has been limited. CD8^+^ MAITs in blood positively correlated with levels of *Akkermansia muciniphila* in biopsy, but only in HIV^-^ non-MSM (**Figures 4A**, **5**). The observation of this relationship only in non-MSM may have been related to CD8^+^ MAITs being depleted in the blood of both HIV^+^ and HIV^-^ MSM cohorts (**Figure 2**). Associations between peripheral MAIT cells and intestinal bacteria may be expected since peripheral MAIT cells traffic to mucosal sites and are influenced by microbial metabolites. Specifically, 5-OP-RU can activate MAIT cells (59), while 6-formylpterin (6-FP) and N-acetyl-6-formylpterin (Ac-6-FP) can inhibit MAIT cell activation (60).

There were also relationships only observed in HIV^+^ and HIV^-^ MSM cohorts and not the HIV^-^ non-MSM. Of particular interest in this category are positive correlations between colonic CD4^+^PD1^+^ T cells and *P. stercorea* in both HIV^+^ and HIV^-^ MSM, and with *Prevotellamassilia timonensis* in HIV^+^ MSM (**Figure 4E**). A relationship between levels of *Prevotella* in MSM and mucosal CD4^+^ T cell exhaustion is of interest because CD4^+^ T cell exhaustion occurs with HIV infection and is linked with disease progression (61), and this effect has been observed in the mucosa (62). To validate some of these findings, we next performed *in vitro* assays to test whether stimulation with cultured bacteria showed expected relationships with correlated immune cell populations, with a focus on MAITs and ILC3s.

### *In vitro* MAIT cell activation reflects *in vivo* microbial associations

First, to confirm MAIT cell frequencies identified by CyTOF analysis, the frequency of MAIT cells in PBMC samples from five individuals of each cohort was determined by conventional flow cytometry. In addition to including the same MAIT cell markers (Va7.2 and CD161) used with the CyTOF analysis, a MAIT-specific tetramer (human MR1) (63) was also used and when compared, values from the CD8^+^MR1^+^ Tetramer and CD8^+^Va7.2^+^CD161^+^ were highly correlated (p<0.0001, r=0.89) (**Supplementary Figure 6A**). Traditional flow cytometry of MAITs with the CD8^+^MR1^+^ Tetramer confirmed our finding from CyTOF of a lower prevalence of MAIT cells in the blood of HIV^+^ and HIV^-^ MSM compared to HIV^-^ non-MSM (**Supplementary Figure 6B**) but only reached statistical significance compared to HIV^+^ MSM (p=0.007). There was a strong correlation (p<0.001, r=0.90) between MAIT cell frequencies determined by Va7.2/CD161 expression from flow cytometry and CyTOF of the same subjects (**Supplementary Figure 6C**).

To test the associations found in the network analysis, peripheral blood mononuclear cells (PBMCs) were cultured with fixed preparations of *Akkermansia muciniphila* (positively associated with MAIT cells). As controls, we selected two of the most negatively associated taxa with MAITs, *Eubacterium ramulus* and *Parabacteroides merdae*, even though they did not fit the rigorous criteria to be included in the network (**Figure 5A**). After 24 hours of stimulation, *A. muciniphila* induced significantly higher CD69 expression on MAIT cells compared to both *E. ramulus* (p = 0.04) and *P. merdae* (p = 0.03) (**Figure 5B**), indicating a functional link between *in vivo* associations and the ability of specific bacterial taxa to activate MAIT cells. Lastly, *A. muciniphilia* supernatant also induced significantly more TNF-α than *E. ramulus* (p=0.03) and *P. merdae* (p=0.03) (**Figure 5B**). These findings confirmed that MAIT cells are decreased in MSM regardless of HIV infection and are activated more readily by bacteria that are associated with *in vivo* MAIT cell frequency than those that are not.

### Bacteria associated with ILC3 frequencies differentially induce colonic ILC3 apoptosis *in vitro*

Because the CyTOF panel did not include additional ILC3 defining markers typically used, namely CD117 and CD294 (CRTH2), ILC3s were identified using the standard markers of CD45^+^Lin^-^CD127^+^CD161^+^CD90^+^ with ICOS used to exclude ILC2s (ICOS^+^). While this panel does not fully exclude ILC1s, ILC3s represent the predominant ILC population in colonic tissues, with ILC1s comprising only approximately 10% (64). To further validate the CyTOF-based panel identification of ILC3s, an expanded ILC3 traditional flow panel, including CD117 and CD294, and the same markers used in the CyTOF panel, were used to stain lamina propria mononuclear cells (LPMC) from colonic resections and analyzed by traditional flow cytometry. Cells stained with both panels showed strong concordance (r = 0.9076, p < 0.0001), confirming the accuracy of the CyTOF gating strategy at identifying ILC3s (**Supplementary Figure 6D**). To test whether bacterial taxa associated with *in vivo* ILC3 frequencies could directly influence ILC3 survival, a panel of bacteria was selected based on positive or negative correlations with colonic ILC3s (**Figure 5C**). *B. fragilis*, *B. adolescentis*, *B. wexlerae* and *P. vulgatus* were positively associated with ILC3 abundance and appeared in the network (**Figure 4**). In contrast, *Parabacteroides distasonis*, *P. copri*, and *Clostridium clostridioforme* showed negative associations with ILC3s with high significance (p < 0.001); although they did not meet the stringent threshold for inclusion in the network. These bacteria were grown under anaerobic conditions, quantified by bicinchoninic acid (BCA) assay, fixed, and then cultured with lamina propria mononuclear cells (LPMCs) isolated from human colonic tissue for 24 hours.

As shown in **Figure 5D**, bacteria positively associated with ILC3s *in vivo* induced little to no ILC3 apoptosis *in vitro*, suggesting potential roles in supporting ILC3 survival or homeostasis. In contrast, negatively associated bacteria triggered markedly increased levels of ILC3 apoptosis, indicating a potential mechanism by which these taxa may contribute to the depletion of ILC3s observed in HIV infection. These findings suggest that microbiome composition can directly modulate the viability of mucosal ILC3s and may contribute to long-term immune dysregulation in the gut.

## Discussion

In this study, we evaluated the relative contributions of HIV infection and MSM-associated factors to immune alterations in the colonic lamina propria and blood. By applying high-dimensional immune profiling to both colonic biopsies and blood, alongside microbiome profiling to colonic biopsy and feces from HIV^+^ MSM, HIV^-^ MSM, and HIV^-^ non-MSM controls, we were able to differentiate differences in both the microbiome and immune cell composition related to HIV infection and MSM identity. This allowed us to explore how host:microbe interactions may be impacted by HIV-associated immune dysfunction or MSM-related factors. Our findings provide three key insights: (1) even after successful viral suppression, HIV infection profoundly alters immune populations in the colonic mucosa, including the depletion of CD4^+^ and CD8^+^ tissue-resident T cells and ILC3s; (2) MSM status/behavior, independent of HIV, is also a factor shaping blood immune profiles, particularly MAIT cells and naïve CD8^+^ T cells; and (3) immune-microbiome networks reveal distinct associations between mucosal immune subsets and bacterial taxa suggesting that HIV-associated immune dysfunction and MSM-associated microbiome differences can effect host:microbe interactions, with functional validation of bacterial effects on MAIT activation and ILC3 viability.

High-dimensional immune profiling using CyTOF as we have previously done (65), allowed us to evaluate 35 immune markers across blood and colonic compartments, confirming that HIV infection is the primary driver of immune cell alterations. The impact of HIV was especially pronounced in the colon, consistent with prior evidence that, even under suppressive ART, immune recovery occurs more slowly in the gut (66). In blood, HIV^+^ MSM exhibit reduced frequencies of CD8^+^CD127^+^ T cells and ILC2s along with increased total CD3^+^ T cells, reflecting skewed T cell dynamics. In the colonic lamina propria, HIV^+^ MSM showed profound losses of CD4^+^CD103^+^ and CD8^+^CD103^+^ tissue-resident T cells as well as marked depletion of ILC3s, highlighting persistent gut-specific immune dysfunction despite successful long-term viral suppression and immune reconstitution as measured by peripheral blood CD4+ count.

CD103 is critical for retention and maintenance of tissue-resident immune cells in mucosal barriers including the lung, gut, and genital tract (67–69). The loss of both CD4^+^ and CD8^+^ CD103^+^ T cells in the colon is likely driven by HIV infection, as tissue-resident T cells are preferentially infected compared to their circulating counterparts (70). Our results build on prior reports of reduced CD103^high^ tissue-resident CD8^+^ T-cells in ART naïve PWH (71) and suggest that this deficit persists despite successful viral suppression and normalization of peripheral blood CD4+ count with ART. Further, decreased CD8^+^CD103^+^ T cells have also been observed in the genital tract of women with HIV (72). One potential driver of the reduction of colonic CD8^+^CD103^+^ T cells is a reduction of CD4^+^ T cells that is driven by HIV infection; CD4^+^ T cells have been shown to be needed for CD8^+^CD103^+^ T cells generation in the lung (73), and positively correlated with CD8^+^CD103^High^ tissue-resident cells in PWH (71). Additionally, another study that observed a reduction in CD103^High^ tissue-resident CD8^+^ T-cells in untreated HIV infection resulted from impaired development due to CD4^+^ T cell loss and increased recruitment of circulating virus-specific CD8^+^ T cells (70). A reduction in colonic CD8^+^ T cells expressing CD103 has been previously linked with other inflammatory states; for instance it was observed in colon and ileum during active untreated Inflammatory Bowel Disease (IBD) and increase with remission (74). Given their role in maintaining mucosal barrier integrity and pathogen surveillance, lower CD103^+^ T cell levels may contribute to the overgrowth of mucosal pathogens such as *Campylobacter*, as observed in this and other studies (9, 15, 33).

ILC3 levels were similarly depleted in PWH compared to both MSM and non-MSM HIV negative controls, consistent with literature reporting that ILC3s fail to recover with ART (44, 75, 76). ILC3s play a critical role in preserving epithelial barrier integrity, regulating mucosal inflammation, and mounting rapid responses to bacterial pathogens through IL-22 and IL-17 production (62). Their selective loss likely contributes to mucosal barrier dysfunction and increased microbial translocation in PWH. Although traditional flow cytometry confirmed the accuracy of our ILC3 identification, the CyTOF panel used here lacked canonical ILC3 markers. Future studies should incorporate additional markers such as RORγt, CD117, and CRTH2 to enhance ILC3 phenotyping.

While HIV was the dominant factor shaping immune composition, MSM status/behavior contributed an additional layer of variation, particularly in mucosal tissues. For example, reductions in blood naïve CD8^+^ T cells and MAIT cells were observed in both HIV^+^ and HIV^-^ MSM compared to HIV^-^ non-MSM controls. These findings, once attributed solely to HIV infection (77), align with recent evidence that MSM-associated factors, likely linked to behavior and associated microbiome differences, can independently modulate immune populations (11, 18, 20). Such observations emphasize the need to control for sexual behaviors among MSM in studies of HIV-related immune changes, particularly in Western cohorts where MSM represents the majority of PWH. Prior reports of MAIT cell depletion in HIV may have been confounded by these factors, as studies often lacked sexual behavior-matched controls. Thus, some prior studies that reported MAIT cell loss in HIV may have been confounded by unaccounted MSM-related differences (78, 79), though MAIT depletion has also been observed in non-MSM cohorts such as HIV-infected children (80) and SIV-infected macaques (81). Mechanistically, MAIT loss is thought to involve migration to mucosal sites and activation-induced cell death, as bacterial exposures like *Escherichia coli* have been shown to trigger MAIT apoptosis *in vitro* (82). Our data suggests that both HIV infection and MSM-associated microbial exposures may contribute to MAIT loss.

Given that sexual behaviors influence the microbiome (83), we also examined associations between behaviors, such as receptive anal intercourse (RAI), partner number, lubricant use, and sexually transmitted infections, with immune alterations. RAI among MSM was associated with increased CD4^+^ and CD8^+^ T cell activation in both blood and colon. Furthermore, having three or more sexual partners within six months correlated with a reduction in central memory T cells. Lubricant use was associated with increased frequencies of colonic CD4^+^, NKT, and CD4^+^PD-1^+^ T cells, suggesting localized mucosal immune activation potentially linked to repeated mechanical or chemical exposure. These patterns suggest that microtrauma from RAI may increase bacterial translocation, while higher partner numbers could lead to repeated bacterial and viral exposures that drive effector T cell differentiation.

We also profiled fecal and colonic microbiome composition using 16S rRNA sequencing. Fecal microbiome differences associated with MSM status were consistent with prior studies, including an enrichment of *Prevotella* and depletion of *Bacteroides* (15). Differences between HIV^+^ and HIV^-^ MSM were also in line with previous findings (28), including a reduction of taxa within the *Lachnospiraceae* and *Ruminococcaceae* families, which are important butyrate producers that support mucosal health (84). Given that mucosal bacteria have potential to interact with the immune system more strongly and thus be impacted by HIV-associated immune dysfunction, we were surprised to detect fewer differences across cohorts with the biopsy compared to fecal microbiome data. This might be because of more heterogeneity in the biopsy microbiome, with some clustering with fecal samples and others having a more unique composition. While all participants underwent the same enema procedure prior to biopsy collection, variability in residual fecal content versus mucosal bacteria may have influenced these findings. It might be advisable to wash biopsy pinches with PBS prior to sequencing to minimize variation due to levels of residual fecal content. However, variability in the degree of clustering of biopsy microbiomes with fecal might also be related to the load of mucosal-adherent bacteria on the biopsy. Many of the highly differentiated biopsy samples of HIV^-^ and HIV^+^ MSM exhibited dominance of a single bacteria in taxa that are known to contain highly mucosal-adherent bacteria, such as adherent-invasive *Escherichia coli* in the Enterobacterales, or various *Campylobacter* and *Helicobacter* spp. in the *Campylobacterales* (85). These taxa also represent potential mucosal pathogens. Pathogens like *Campylobacter jejuni* and *Shigella* are typically transmitted via fecal-oral routes but are known to be sexually transmitted among MSM (86).

We further explored behavioral correlations with microbiome composition and found that *M. massiliensis*, *C. catus*, and *C. disporicum* were reduced with RAI. While *C. catus*, a butyrate producer (87), may benefit barrier function, *C. disporicum* has been implicated in opportunistic infections (88). We also observed that *C. catus* and *O.* sp*lanchnicus* which both have anti-inflammatory properties (89) were reduced in participants with 3 or more sexual partners, whereas *P. harei*, a potential opportunistic pathogen (90), was enriched.

Our network analysis focused on identifying relationships between immune cell populations and microbial species that differed by HIV infection or MSM status. The resulting immune-microbe network revealed extensive connectivity between immune cell populations and microbial taxa, reflecting the complexity of host-microbiome interactions. Most associations occurred between colonic immune populations and microbes from the same tissue or feces, underscoring the local nature of many of these interactions.

Intriguing and functionally relevant associations were with colonic ILC3s and peripheral MAIT cells. Both cell types are mucosal-focused, microbe-responsive populations implicated in maintaining gut barrier integrity and antimicrobial defense (91, 92). Given their well-established sensitivity to microbial cues, we prioritized these populations for *in vitro* validation using bacterial taxa that were positively or negatively associated with their frequencies *in vivo*. Associations between peripheral MAIT cells and intestinal bacteria may be expected since peripheral MAIT cells traffic to mucosal sites and are influenced by microbial metabolites. Specifically, 5-OP-RU, a metabolite in the riboflavin biosynthesis pathway, can activate MAIT cells (59), while 6-formylpterin (6-FP) and N-acetyl-6-formylpterin (Ac-6-FP) can inhibit MAIT cell activation (60). Although *A. muciniphila* can produce 5-OP-RU and its supernatant did stimulate MAITs *in vitro* more than negatively correlated controls, more study is needed to explore the driving factors, as riboflavin biosynthesis is common in the microbiome and most riboflavin-producers did not significantly correlate with MAITs. The four tested bacteria that positively associated with ILC3 frequency induced significantly less apoptosis than the two tested negatively correlated controls. These functional results provide direct evidence that specific members of the gut microbiota may promote or disrupt mucosal immune cell viability and activation, supporting a mechanistic basis for the immune-microbial correlations observed *in vivo*.

While our integrative study design and analysis provides new insights into host–microbe interactions in MSM with and without HIV, several limitations warrant mention. First, behavioral data were self-reported and may be subject to recall bias and although participants with recent antibiotic use were excluded, data on ART Pre-exposure prophylaxis (PrEP) use, diet, and substance use were not uniformly available. These unmeasured factors could influence gut microbial composition and immune parameters and should be incorporated in future longitudinal and interventional studies. Second, although the CyTOF panel was extensive, additional ILC3-defining markers (e.g., RORγt, CD117, CRTH2) could further refine subset populations. It is also noted that our *in vitro* assays do not fully replicate the complexity of the mucosal niche. Future longitudinal and interventional studies, including controlled trials assessing behavioral or microbiome-targeted modulation, will be essential to establish mechanistic links between specific taxa, mucosal immune cell dynamics, and HIV persistence.

Overall, this study provides a detailed view of how HIV infection and MSM-associated microbiome/behaviors jointly shape gastrointestinal immunity and microbiome composition. By integrating high-dimensional immune and microbiome profiling with functional validation, we delineate immune alterations attributable to HIV versus those linked to MSM behavior. These findings underscore the need to control for sexual behavior when examining immune dysfunction in PWH and suggest that behavioral and microbial factors can impact mucosal immunity even in the absence of HIV. This research advances our understanding of the multifaceted relationships among behavior, gut microbial communities, and immunity. Future studies should build on these insights to understand the role of these relationships in driving detrimental immunologic changes, such as inflammation, in the MSM community, with or without HIV, and for the greater population of PWH.

## Methods

### Study population

**Table 1** contains complete demographic information for the study participants. The samples were collected as part of three different studies that were reviewed and approved by the Colorado Multiple Institutional Review Board (CoMIRB 14-1595, 15-1692, 17-1512). Informed consent was obtained from all participants. Information on the fecal microbiome of some of these individuals was previously published (3). Individuals were divided into 3 cohorts (1) 33 chronic HIV infection on long-term ART (HIV^+^ MSM): ART for ≥ 12 months with a minimum of three ART drugs prior to study entry and plasma HIV RNA < 50 copies/mL within 30 days prior to study entry and no plasma HIV RNA ≥ 50 copies/mL in the past 6 months. Mean values of CD4+ T cell counts were high at 727 per mL of blood and viral load mean was 5.73. Only men were included and 32 were confirmed MSM and one bi-sexual based on a sexual behavior questionnaire. (2) 16 HIV seronegative MSM (HIV^-^ MSM) were defined as high risk for contracting HIV based on criteria used in a prior study of a candidate HIV vaccine: (a) a history of unprotected anal intercourse with one or more male or male-to-female transgender partners, (b) anal intercourse with two or more male or male-to-female transgender partners, or (c) being in a sexual relationship with a person who has been diagnosed with HIV (93) and (3) 21 HIV seronegative non-MSM (HIV^-^ non-MSM). Individuals who reported taking antibiotics within 3 months of sample collection were excluded. No statistically significant differences were observed with Bristol Stool Scale between the cohorts.

### Sample collection

#### Feces collection

Study participants collected a fecal sample using a commode specimen collector. Fecal samples were stored at -4°C during transport (for <48 hours), aliquoted and transferred to -80°C upon delivery to the lab.

#### Biopsy collection

Participants were given two Fleet saline enemas followed by flexible sigmoidoscopy with collection of 30 pinch biopsies from the colorectal tissue using 2.4 mm forceps. Four pinch biopsies were put into 0.25 mL of RNALater for microbiome (16S rRNA) analysis. The remaining biopsies were put in 10 mL low barium Phosphate Buffer Solution (PBS) for use with CyTOF and traditional flow cytometry. The biopsies were digested for 1.5 hours with collagenase (1 mg/mL) and DNAse (5 uL/mL) as previously described (13). Pinches were then mashed and filtered through a 70-micron nylon cell strainer and washed with 15 mL low barium PBS, centrifuged and resuspended in 2 mL low barium PBS. Equal amounts of cells were divided and immediately stained for CyTOF or traditional flow cytometry.

#### Blood collection

Blood was collected by venipuncture into EDTA tubes and peripheral blood mononuclear cells (PBMC) were isolated by Ficol density gradient centrifugation as previously described (94).

### CyTOF staining

PBMC and pinch biopsy single cell suspensions were stained for live–dead cell distinction using 2.5 µM Cisplatin (Fluidigm, South San Francisco, CA, USA). Cells were re-suspended in 65 µl barium free FACS buffer (low barium PBS with 0.1% BSA and 2 mM EDTA) and incubated for 30 min at 4°C with a 35 µl cocktail of metal-conjugated antibodies (1 µl each) (**Supplementary Table 1**). Cells were washed and resuspended with MaxPar fix with DNA intercalator (0.125 µM Iridium-191/193; Fluidigm, South San Francisco, CA, USA) and EQ Four Element Calibration Beads (Fluidigm, South San Francisco, CA, USA) were added. Cells were acquired using a CyTOF2 mass cytometer (Fluidigm, South San Francisco, CA, USA), CyTOF software v.6.0.626 with noise reduction, a lower convolution threshold of 200, event length limits of 10–150 pushes, a sigma value of 3, and a flow rate of 0.045 ml/min. Runs were concatenated using the FCS file concatenation tool from Cytobank and normalized using the EQ Four calibration beads. For our gating strategy we used high confidence immune populations that did not express the marker of interest or that had definitive bimodal staining to define marker positivity of other immune cell subsets based on careful, blinded manual gating. These gates were then directly applied to the population of interest. The monoclonal antibody (mAb) panel (**Supplementary Table 1**) targeted a wide variety of immune populations with a focus on CD4^+^ and CD8^+^ T cells, including markers for T regulatory cells (Tregs), Th17, Th1, Tfh cells, T cell maturation, inhibitory receptors, gut homing receptors, markers of acute/chronic activation, and Mucosal Invariant T Cells (MAITs). The panel also identified populations of monocytes, macrophages, dendritic cells, B cells, Natural Killer (NK) cells, and NK T cells for a total of 49 populations in the blood and 48 in the biopsy. Participant blinded analysis focused on these immune populations because they had distinct or bimodal staining patterns which ensured accurate and reproducible gating. A representative gating strategy on blood immune populations is shown in **Supplementary Figure 1**.

### Mucosal-associated invariant T-cell immunofluorescence staining and *in vitro* stimulation

The frequency of MAIT cells was determined by surface staining. PBMCs (2–3 x 10^6^ cells) were stained for MAIT cell identification using MR1 tetramer at room temperature for 40 minutes. After washing cells were stained with Live/Dead Fixable Aqua Stain Kit (Invitrogen L34966) per the manufacturer’s protocol. Cells were surface stained for CD3 (BD Biosciences 563800), CD4 (BD Biosciences 560158), CD8 (Biolegend 344744), CD161 (Biolegend 339908), and Vα7.2 (Biolegend 351720) at 4°C for 30 minutes after washing. Cells were washed and fixed with 1.6% PFA buffer and stored at 4°C until ready for flow. Cells were analyzed on a BD LSRFortessa flow cytometer (BD Immunocytometry Systems), and at least 500,000 events were collected. Antibody capture beads (BD Biosciences) were used to perform electronic compensation. Beads were stained separately with individual antibodies. Data were analyzed using FlowJo software. Singlets were gated by their forward scatter profile. Lymphocytes were gated by their side scatter and forward scatter profile. Live, CD3^+^, CD8^+^ cells were selected. MAIT cell frequency was analyzed using CD8 and MR1 tetramer double positive population, or a CD161 and Vα7.2 double positive population. Bi-exponential scaling was used in all dot plots, and all the populations analyzed consisted of over 100 events to ensure an adequate number of events. FMO populations were used for Vα7.2 and CD161. The frequency of activated cytokine producing MAIT cells was determined by intracellular cytokine staining. PBMC’s (2 x 10^6^ cells) were cultured in complete media (89% RPMI, 10% Heat-inactivated human serum, 1% pen-strep glutamine) in 5 ml polypropylene tubes. Cells were stimulated with fixed *A. muciniphila* (DSM 22959, grown in BHI + mucin media, Duerkop Lab), *E. ramulus* (DSM 15684, grown in Chopped Meat + Carbohydrate media, Anaerobe Systems), or *P. merdae* (DSM 19495, grown in Chopped Meat media, Anaerobe System) at a final protein concentration of 32 µg/µl. Bacteria were fixed in 2% paraformaldehyde for 20 minutes at room temperature, washed twice with sterile PBS, and stored at 4°C until use in stimulation assays. Bacteria was normalized to 436 µg/µl using Pierce BCA Protein Assay Kit (Thermo Scientific 23225). Cells were incubated for 6 hours or 28 hours at 37°C in a humidified 5% CO2 atmosphere and a 5-degree slant with Brefeldin a (BD biosciences 555029) added for the final 4 hours of incubation. Cells were stained with Live/Dead Fixable Aqua Dead Cell Stain Kit (Invitrogen L34957) following manufacturers protocol. Cells were surface stained with: anti-CD3 (BD Biosciences 563800), anti-CD4 (BD Biosciences 560158), anti-CD25 (BD Biosciences 555432), anti-CD69 (BD Biosciences 555530), anti-Vα7.2 (Biolegend 351720), anti-CD161 (Biolegend 339908) for 30 minutes at 4°C. Cells were fixed using Fix and Perm medium (Invitrogen GAS004) overnight at 4°C. Cells were permeabilized using Fix and Perm medium (Invitrogen GAS004) and intracellular stained with: anti-IFNγ (BD Biosciences 562988) and anti-TNFα (Biolegend 502930) antibodies for 2 hours at 4°C. Cells were washed and fixed with 1.6% paraformaldehyde. Flow cytometry was run immediately after fixation on a BD LSRFortessa.

### ILC3 immunophenotyping and *in vitro* bacterial stimulation for apoptosis assay

The frequency and viability of group 3 innate lymphoid cells (ILC3s) were assessed by surface staining and flow cytometry following bacterial stimulation. Lamina propria mononuclear cells (LPMCs) were isolated from human colonic biopsies using enzymatic digestion (collagenase IV and DNase I) and mechanical disruption. Isolated LPMCs (1–2 × 10^6^ cells) were cultured in complete RPMI media (89% RPMI 1640, 10% heat-inactivated fetal bovine serum, 1% penicillin-streptomycin-glutamine) in 48-well flat-bottom plates (Falcon).

To validate the identification of ILC3s using a CyTOF-compatible gating strategy, LPMCs were stained with a comprehensive surface marker panel that included both conventional and CyTOF-based ILC3 markers. The panel consisted of anti-CD45 (BD Biosciences 563204), anti-CD127 (BD Biosciences 563225), anti-CD161 (Biolegend 339918), anti-CD90 (BD Biosciences 563070), and anti-ICOS (BD Biosciences 562833), along with a Lineage cocktail composed of antibodies against CD3, CD19, CD20, CD56 (Biolegend 363601). Additional traditional ILC3 markers included anti-CD117 (Biolegend 313217) and anti-CRTH2 (Biolegend 350113). Cells were first gated as ILC3s using the CyTOF-equivalent phenotype (CD45^+^Lin^-^CD127^+^CD161^+^ICOS^-^CD90^+^), and then re-gated using a traditional ILC3 definition (CD45^+^Lin^-^CD127^+^ CD161^+^ CD117^+^ CRTH2^-^). Frequencies of ILC3s obtained by each gating approach were calculated as a percentage of live, CD45^+^Lin^-^ cells and were compared by Pearson correlation analysis.

Fixed bacteria were prepared from species found to be either positively or negatively associated with ILC3 frequency *in vivo*. These included *B. fragilis* (DSM 2151, grown in Mega media), *B. adolescentis* (DSM 20083, Mega media), *P. vulgatus* (DSM 1447, YCFA [Yeast, Casitone, Fatty Acids] media), and *B. wexlerae* (DSM 19850, Mega media) (positively associated), and *P. distasonis* (DSM 18205, YCFA media), and *C. clostrdioforme* (DSM 933, Mega media) (negatively associated). Bacterial cultures were grown under anaerobic conditions, washed, and fixed with 2% paraformaldehyde for 20 minutes at room temperature. Protein concentration was quantified using the Pierce BCA Protein Assay Kit (Thermo Scientific 23225) and normalized to a final concentration of 20 µg/µl per well. Cells were incubated with fixed bacteria for 24 hours at 37 °C in a humidified 5% CO_2_ incubator and then stained for Annexin V and Propidium iodide (PI) (Thermo Fisher Scientific V13241) following manufacturer’s instructions.

ILC3s were identified as CD45^+^Lin^-^CD127^+^CD161^+^ CD117^+^CRTH2^-^ cells. Apoptotic cells were defined by positive staining with Annexin V and PI. Fluorescence-minus-one (FMO) controls were used to define gating thresholds, and populations with fewer than 100 events were excluded from analysis. Data were analyzed using FlowJo software (Tree Star, Inc.), and bi-exponential scaling was applied to all dot plots.

### Generation of microbiome data

DNA was extracted from fecal samples and biopsy using the PowerSoil kit (Qiagen Cat. 47014). Extracted DNA was PCR amplified with barcoded primers targeting the V4 region of 16S rRNA gene according to the Earth Microbiome Project 16S Illumina Amplicon protocol with the 515F:806R primer constructs as detailed in (95). Each PCR product was quantified using PicoGreen (Invitrogen), and equal amounts (ng) of DNA from each sample were pooled and cleaned using the UltraClean PCR Clean-Up kit (MoBio). Pooled DNA was sequenced on a MiSeq personal sequencer. Dada2 (49) was used to denoise and bin identical sequences into ASVs, which were assigned taxonomically using the RDP classifier trained on the greengenes2 (96) taxonomy (97) using QIIME 2 version 04.2021 (98). A phylogenetic tree was built using the SEPP plugin (99). Features that did not classify at the phylum level or were classified as mitochondria or chloroplast were filtered from the analysis. For integrated analyses (described below), ASVs were assigned using BLASTN 2.13.0+ (100).

### Data analysis

#### Analysis of immune cell populations

Differences in immune populations were identified by cohort, after accounting for age, day of sample collection, and gender. A full model of analyte ~ age + day + gender + cohort were compared to a reduced model of analyte ~ age + day + gender. Day refers to the relative day of collection across the course of the study (range 1-1457) and is included to account for possible batch effects associated with running each sample when it was fresh. Within each population, pairwise significant differences across the cohort term in the model were using Tukey’s HSD to correct for multiple comparisons, setting the significance level to 0.05. For each immune population that was measured in both blood and biopsy, differences across tissues were evaluated with a paired T test, with p-values adjusted for multiple comparisons by FDR. Significance of MSM and HIV infection status on pairwise distances between samples was calculated using the adonis test (101).

### Microbiome analysis

Beta diversity was evaluated using both unweighted (47) and weighted (48) UniFrac, with ordination using PCoA using QIIME 2 version 04.2021 (98). Assessments of significant associations with PERMANOVA or Adonis using QIIME 2 version 04.2021. The data was rarefied at a depth of 17401 for fecal samples only data, and 5458 for biopsy only and biopsy with fecal data sequences per sample prior to the application of UniFrac. Differences by cohort were identified using unrarefied data but correcting for sequencing depth in the model. We first filtered ASVs to include only those having >= 14 (20% of 70) non-zero samples per tissue (feces, colonic). We then created linear models of the form relative abundance ~ cohort + reads. Reads represented the number of sequences per sample. We adjusted the p-values on the overall regression models (F statistic) using FDR, adjusting separately for feces and biopsy. We computed pairwise significant differences across the cohorts using Tukey’s HSD correction for multiple comparisons, setting the significance level to 0.05 (**Supplementary Table 4**).

### Integrative analyses

An integrated data set was created consisting of immune cell subsets measured in blood (number of analytes n=49), immune cell subsets measured in colonic biopsy (n=48), ASV frequencies measured in feces (n=163), and ASV frequencies measured in colonic biopsy (p=122). Immune cell subsets are reported as frequency (percent of parent population) unless otherwise noted such as in **Supplementary Figure 2**. Microbiome data is reported as arcsinh-transformed parts per hundred (log(x + sqrt(x^2^ + 1))). Only ASVs present in at least 24 samples were included in the analysis. When multiple ASVs were assigned to the same taxa, we added a trailing sequence number for uniqueness (e.g. g_Bacteroides.s_uniformis.43). To better characterize the ASVs, we submitted FASTA sequences to NCBI’s BLASTn tool (102). For sequences with > 97% identity, we used the BLAST scientific name in figures. If more than one result was returned with the same % identity, we used the name from the first result.

### Network analysis

Pairwise linear regressions were performed across all analytes from 5 pairs of samples (blood cells:colonic cells, blood cells:colonic microbes, blood cells:fecal microbes, colonic cells:colonic microbes, and colonic cells:fecal microbes). The resulting networks of associated microbes and immune cell populations visualized using the bioinformatics tool VOLARE (52). The full regression model was of the form analyte1 ~ age + day + gender + cohort + analyte2 + analyte2 x cohort, while the reduced model was of the form analyte1 ~ age + day + gender + cohort. If one of the analytes was a microbe, the number of reads was added to the models (e.g. analyte1 ~ reads + age + day + gender + cohort + analyte2 + analyte2 x cohort). The resulting p-values were adjusted from the partial F-test using FDR, correcting separately for each assay pair. P-values comparing each slope to 0 were also computed. The final network was limited to those relationships with an FDR-adjusted p-value < 0.05 and the p-value on at least one of the slopes < 0.005. Edge inclusion followed an iterative process balancing interpretability with statistical rigor. Relationships were retained if the overall model passed an FDR-adjusted p < 0.05 (partial F-test) and at least one cohort-specific slope had p < 0.005. To ensure robustness, we excluded associations with difference in fit(s) (DFFITS) > 2 or skew > 3 to remove outlier-driven results. Results were reported for ASVs which provide a more precise taxonomic level and produced more significant edges connected to microbes (31) than the genera analysis (16).

### Analysis of immune cell populations and microbes differing by behavior

Data from 5 questions, answered by 10 to 39 HIV^-^ MSM and HIV^+^ MSM study participants, was analyzed. For each question, for each immune population, a full model of the form analyte ~ HIV + answer was compared to a reduced model of the form analyte ~ HIV. For each microbe, a full model of the form analyte ~ HIV + reads + answer was compared to a reduced model of the form analyte ~ HIV + reads. The resulting partial F statistic was used to identify models in which the full model was significantly better than the reduced model (p < 0.05). No correction for multiple comparisons was made due to the exploratory nature of this analysis.

Overall, linear models were created in the R statistical computing environment with the method lm. Analyses for differences in means across cohorts were supported with the method glht from the package multcomp. The network graph was created with the R package igraph.

## Data Availability

Microbiome data are available at EBI (https://www.ebi.ac.uk) under accession number ERP179699.

## References

[1] Chun TW Nickle DC Justement JS Meyers JH Roby G Hallahan CW . Persistence of HIV in gut-associated lymphoid tissue despite long-term antiretroviral therapy. J Infect Dis. (2008) 197:714–20. doi: 10.1086/527324, PMID: 18260759

[2] Zevin AS McKinnon L Burgener A Klatt NR . Microbial translocation and microbiome dysbiosis in HIV-associated immune activation. Curr Opin HIV AIDS. (2016) 11:182–90. doi: 10.1097/COH.0000000000000234, PMID: 26679414 PMC4752849

[3] Armstrong AJS Quinn K Fouquier J Li SX Schneider JM Nusbacher NM . . doi: 10.1128/mSystems.01178-20, PMID: 34006628 PMC8269254

[4] Brenchley JM Schacker TW Ruff LE Price DA Taylor JH Beilman GJ . CD4+ T cell depletion during all stages of HIV disease occurs predominantly in the gastrointestinal tract. J Exp Med. (2004) 200:749–59. doi: 10.1084/jem.20040874, PMID: 15365096 PMC2211962

[5] Guadalupe M Reay E Sankaran S Prindiville T Flamm J McNeil A . Severe CD4+ T-cell depletion in gut lymphoid tissue during primary human immunodeficiency virus type 1 infection and substantial delay in restoration following highly active antiretroviral therapy. J Virol. (2003) 77:11708–17. doi: 10.1128/jvi.77.21.11708-11717.2003, PMID: 14557656 PMC229357

[6] Estes JD Harris LD Klatt NR Tabb B Pittaluga S Paiardini M . Damaged intestinal epithelial integrity linked to microbial translocation in pathogenic simian immunodeficiency virus infections. PloS Pathog. (2010) 6:e1001052. doi: 10.1371/journal.ppat.1001052, PMID: 20808901 PMC2924359

[7] Shah SV Manickam C Ram DR Reeves RK . Innate Lymphoid Cells in HIV/SIV Infections. Front Immunol. (2017) 8:1818. doi: 10.3389/fimmu.2017.01818, PMID: 29326704 PMC5733347

[8] Dillon SM Lee EJ Kotter CV Austin GL Dong Z Hecht DK . An altered intestinal mucosal microbiome in HIV-1 infection is associated with mucosal and systemic immune activation and endotoxemia. Mucosal Immunol. (2014) 7:983–94. doi: 10.1038/mi.2013.116, PMID: 24399150 PMC4062575

[9] Vujkovic-Cvijin I Dunham RM Iwai S Maher MC Albright RG Broadhurst MJ . Dysbiosis of the gut microbiota is associated with HIV disease progression and tryptophan catabolism. Sci Transl Med. (2013) 5:193ra191. doi: 10.1126/scitranslmed.3006438, PMID: 23843452 PMC4094294

[10] van Unen V Li N Molendijk I Temurhan M Hollt T van der Meulen-de Jong AE . Mass Cytometry of the Human Mucosal Immune System Identifies Tissue- and Disease-Associated Immune Subsets. Immunity. (2016) 44:1227–39. doi: 10.1016/j.immuni.2016.04.014, PMID: 27178470

[11] Verboeket SO Wit FW Verheij E van Zoest RA Kootstra NA van der Valk M . Human Immunodeficiency Virus (HIV)-Negative Men Who Have Sex With Men Have Higher CD8+ T-Cell Counts and Lower CD4+/CD8+ T-Cell Ratios Compared With HIV-Negative Heterosexual Men. J Infect Dis. (2021) 224:1187–97. doi: 10.1093/infdis/jiaa048, PMID: 32003801 PMC8514179

[12] Kelley CF Kraft CS de Man TJ Duphare C Lee HW Yang J . The rectal mucosa and condomless receptive anal intercourse in HIV-negative MSM: implications for HIV transmission and prevention. Mucosal Immunol. (2017) 10:996–1007. doi: 10.1038/mi.2016.97, PMID: 27848950 PMC5433931

[13] Li SX Sen S Schneider JM Xiong KN Nusbacher NM Moreno-Huizar N . Gut microbiota from high-risk men who have sex with men drive immune activation in gnotobiotic mice and in vitro HIV infection. PloS Pathog. (2019) 15:e1007611. doi: 10.1371/journal.ppat.1007611, PMID: 30947289 PMC6448819

[14] Huang KD Amend L Galvez EJC Lesker TR de Oliveira R Bielecka A . Establishment of a non-Westernized gut microbiota in men who have sex with men is associated with sexual practices. Cell Rep Med. (2024) 5:101426. doi: 10.1016/j.xcrm.2024.101426, PMID: 38366600 PMC10982974

[15] Armstrong AJS Shaffer M Nusbacher NM Griesmer C Fiorillo S Schneider JM . An exploration of Prevotella-rich microbiomes in HIV and men who have sex with men. Microbiome. (2018) 6:198. doi: 10.1186/s40168-018-0580-7, PMID: 30396369 PMC6219090

[16] Vujkovic-Cvijin I Sortino O Verheij E Sklar J Wit FW Kootstra NA . HIV-associated gut dysbiosis is independent of sexual practice and correlates with noncommunicable diseases. Nat Commun. (2020) 11:2448. doi: 10.1038/s41467-020-16222-8, PMID: 32415070 PMC7228978

[17] Haaland RE Fountain J Hu Y Holder A Dinh C Hall L . Repeated rectal application of a hyperosmolar lubricant is associated with microbiota shifts but does not affect PrEP drug concentrations: results from a randomized trial in men who have sex with men. J Int AIDS Soc. (2018) 21:e25199. doi: 10.1002/jia2.25199, PMID: 30378274 PMC6207839

[18] Kelley CF Pollack I Yacoub R Zhu Z Van Doren VE Gumber S . Condomless receptive anal intercourse is associated with markers of mucosal inflammation in a cohort of men who have sex with men in Atlanta, Georgia. J Int AIDS Soc. (2021) 24:e25859. doi: 10.1002/jia2.25859, PMID: 34911162 PMC8673926

[19] Neff CP Krueger O Xiong K Arif S Nusbacher N Schneider JM . Fecal Microbiota Composition Drives Immune Activation in HIV-infected Individuals. EBioMedicine. (2018) 30:192–202. doi: 10.1016/j.ebiom.2018.03.024, PMID: 29650491 PMC5952409

[20] Yamada E Martin CG Moreno-Huizar N Fouquier J Neff CP Coleman SL . Intestinal microbial communities and Holdemanella isolated from HIV+/- men who have sex with men increase frequencies of lamina propria CCR5(+) CD4(+) T cells. Gut Microbes. (2021) 13:1997292. doi: 10.1080/19490976.2021.1997292, PMID: 34818131 PMC8632320

[21] Hladik F McElrath MJ . Setting the stage: host invasion by HIV. Nat Rev Immunol. (2008) 8:447–57. doi: 10.1038/nri2302, PMID: 18469831 PMC2587276

[22] Sullivan PS Salazar L Buchbinder S Sanchez TH . Estimating the proportion of HIV transmissions from main sex partners among men who have sex with men in five US cities. Aids. (2009) 23:1153–62. doi: 10.1097/QAD.0b013e32832baa34, PMID: 19417579

[23] Bleul CC Wu L Hoxie JA Springer TA Mackay CR . The HIV coreceptors CXCR4 and CCR5 are differentially expressed and regulated on human T lymphocytes. Proc Natl Acad Sci U.S.A. (1997) 94:1925–30. doi: 10.1073/pnas.94.5.1925, PMID: 9050881 PMC20019

[24] Choe H Farzan M Sun Y Sullivan N Rollins B Ponath PD . The beta-chemokine receptors CCR3 and CCR5 facilitate infection by primary HIV-1 isolates. Cell. (1996) 85:1135–48. doi: 10.1016/s0092-8674(00)81313-6, PMID: 8674119

[25] Noguera-Julian M Rocafort M Guillen Y Rivera J Casadella M Nowak P . Gut Microbiota Linked to Sexual Preference and HIV Infection. EBioMedicine. (2016) 5:135–46. doi: 10.1016/j.ebiom.2016.01.032, PMID: 27077120 PMC4816837

[26] Coleman SL Neff CP Li SX Armstrong AJS Schneider JM Sen S . Can gut microbiota of men who have sex with men influence HIV transmission? Gut Microbes. (2020) 11:610–9. doi: 10.1080/19490976.2019.1700756, PMID: 32036739 PMC7524317

[27] Chen Y Lin H Cole M Morris A Martinson J McKay H . Signature changes in gut microbiome are associated with increased susceptibility to HIV-1 infection in MSM. Microbiome. (2021) 9:237. doi: 10.1186/s40168-021-01168-w, PMID: 34879869 PMC8656045

[28] Vujkovic-Cvijin I Somsouk M . HIV and the Gut Microbiota: Composition, Consequences, and Avenues for Amelioration. Curr HIV/AIDS Rep. (2019) 16:204–13. doi: 10.1007/s11904-019-00441-w, PMID: 31037552 PMC6579656

[29] Tincati C Douek DC Marchetti G . Gut barrier structure, mucosal immunity and intestinal microbiota in the pathogenesis and treatment of HIV infection. AIDS Res Ther. (2016) 13:19. doi: 10.1186/s12981-016-0103-1, PMID: 27073405 PMC4828806

[30] Sorini C Cardoso RF Gagliani N Villablanca EJ . Commensal Bacteria-Specific CD4(+) T Cell Responses in Health and Disease. Front Immunol. (2018) 9:2667. doi: 10.3389/fimmu.2018.02667, PMID: 30524431 PMC6256970

[31] Zilberman-Schapira G Zmora N Itav S Bashiardes S Elinav H Elinav E . The gut microbiome in human immunodeficiency virus infection. BMC Med. (2016) 14:83. doi: 10.1186/s12916-016-0625-3, PMID: 27256449 PMC4891875

[32] Zicari S Sessa L Cotugno N Ruggiero A Morrocchi E Concato C . doi: 10.3390/v11030200, PMID: 30818749 PMC6466530

[33] Lozupone CA Li M Campbell TB Flores SC Linderman D Gebert MJ . Alterations in the gut microbiota associated with HIV-1 infection. Cell Host Microbe. (2013) 14:329–39. doi: 10.1016/j.chom.2013.08.006, PMID: 24034618 PMC3864811

[34] Lu W Feng Y Jing F Han Y Lyu N Liu F . Association Between Gut Microbiota and CD4 Recovery in HIV-1 Infected Patients. Front Microbiol. (2018) 9:1451. doi: 10.3389/fmicb.2018.01451, PMID: 30034377 PMC6043814

[35] Mutlu EA Keshavarzian A Losurdo J Swanson G Siewe B Forsyth C . A compositional look at the human gastrointestinal microbiome and immune activation parameters in HIV infected subjects. PloS Pathog. (2014) 10:e1003829. doi: 10.1371/journal.ppat.1003829, PMID: 24586144 PMC3930561

[36] Ivanov II Atarashi K Manel N Brodie EL Shima T Karaoz U . Induction of intestinal Th17 cells by segmented filamentous bacteria. Cell. (2009) 139:485–98. doi: 10.1016/j.cell.2009.09.033, PMID: 19836068 PMC2796826

[37] Nava GM Friedrichsen HJ Stappenbeck TS . Spatial organization of intestinal microbiota in the mouse ascending colon. Isme J. (2011) 5:627–38. doi: 10.1038/ismej.2010.161, PMID: 20981114 PMC3105732

[38] Thome JJ Yudanin N Ohmura Y Kubota M Grinshpun B Sathaliyawala T . Spatial map of human T cell compartmentalization and maintenance over decades of life. Cell. (2014) 159:814–28. doi: 10.1016/j.cell.2014.10.026, PMID: 25417158 PMC4243051

[39] Kumar BV Ma W Miron M Granot T Guyer RS Carpenter DJ . Human Tissue-Resident Memory T Cells Are Defined by Core Transcriptional and Functional Signatures in Lymphoid and Mucosal Sites. Cell Rep. (2017) 20:2921–34. doi: 10.1016/j.celrep.2017.08.078, PMID: 28930685 PMC5646692

[40] Senda T Dogra P Granot T Furuhashi K Snyder ME Carpenter DJ . Microanatomical dissection of human intestinal T-cell immunity reveals site-specific changes in gut-associated lymphoid tissues over life. Mucosal Immunol. (2019) 12:378–89. doi: 10.1038/s41385-018-0110-8, PMID: 30523311 PMC6375790

[41] Szabo PA Miron M Farber DL . Location, location, location: Tissue resident memory T cells in mice and humans. Sci Immunol. (2019) 4. doi: 10.1126/sciimmunol.aas9673, PMID: 30952804 PMC6778482

[42] Sathaliyawala T Kubota M Yudanin N Turner D Camp P Thome JJ . Distribution and compartmentalization of human circulating and tissue-resident memory T cell subsets. Immunity. (2013) 38:187–97. doi: 10.1016/j.immuni.2012.09.020, PMID: 23260195 PMC3557604

[43] Lyu Y Zhou Y Shen J . An Overview of Tissue-Resident Memory T Cells in the Intestine: From Physiological Functions to Pathological Mechanisms. Front Immunol. (2022) 13:912393. doi: 10.3389/fimmu.2022.912393, PMID: 35711464 PMC9192946

[44] Wang Y Lifshitz L Gellatly K Vinton CL Busman-Sahay K McCauley S . HIV-1-induced cytokines deplete homeostatic innate lymphoid cells and expand TCF7-dependent memory NK cells. Nat Immunol. (2020) 21:274–86. doi: 10.1038/s41590-020-0593-9, PMID: 32066947 PMC7044076

[45] Koprivica I Stanisavljevic S Micanovic D Jevtic B Stojanovic I Miljkovic D . ILC3: a case of conflicted identity. Front Immunol. (2023) 14:1271699. doi: 10.3389/fimmu.2023.1271699, PMID: 37915588 PMC10616800

[46] Guo X Fu YX . The tragic fate of group 3 innate lymphoid cells during HIV-1 infection. J Clin Invest. (2015) 125:3430–2. doi: 10.1172/JCI83823, PMID: 26301808 PMC4588309

[47] Lozupone C Knight R . UniFrac: a new phylogenetic method for comparing microbial communities. Appl Environ Microbiol. (2005) 71:8228–35. doi: 10.1128/AEM.71.12.8228-8235.2005, PMID: 16332807 PMC1317376

[48] Lozupone CA Hamady M Kelley ST Knight R . Quantitative and qualitative beta diversity measures lead to different insights into factors that structure microbial communities. Appl Environ Microbiol. (2007) 73:1576–85. doi: 10.1128/AEM.01996-06, PMID: 17220268 PMC1828774

[49] Callahan BJ McMurdie PJ Rosen MJ Han AW Johnson AJ Holmes SP . DADA2: High-resolution sample inference from Illumina amplicon data. Nat Methods. (2016) 13:581–3. doi: 10.1038/nmeth.3869, PMID: 27214047 PMC4927377

[50] Atarashi K Tanoue T Shima T Imaoka A Kuwahara T Momose Y . Induction of colonic regulatory T cells by indigenous Clostridium species. Science. (2011) 331:337–41. doi: 10.1126/science.1198469, PMID: 21205640 PMC3969237

[51] Eckburg PB Bik EM Bernstein CN Purdom E Dethlefsen L Sargent M . Diversity of the human intestinal microbial flora. Science. (2005) 308:1635–8. doi: 10.1126/science.1110591, PMID: 15831718 PMC1395357

[52] Siebert JC Neff CP Schneider JM Regner EH Ohri N Kuhn KA . VOLARE: visual analysis of disease-associated microbiome-immune system interplay. BMC Bioinf. (2019) 20:432. doi: 10.1186/s12859-019-3021-0, PMID: 31429723 PMC6701114

[53] Zhang W Zhou Q Liu H Xu J Huang R Shen B . Bacteroides fragilis strain ZY-312 facilitates colonic mucosa regeneration in colitis via motivating STAT3 signaling pathway induced by IL-22 from ILC3 secretion. Front Immunol. (2023) 14:1156762. doi: 10.3389/fimmu.2023.1156762, PMID: 37114045 PMC10126674

[54] Bao B Wang Y Boudreau P Song X Wu M Chen X . Bacterial Sphingolipids Exacerbate Colitis by Inhibiting ILC3-derived IL-22 Production. Cell Mol Gastroenterol Hepatol. (2024) 18:101350. doi: 10.1016/j.jcmgh.2024.04.007, PMID: 38704148 PMC11222953

[55] Qi X Yun C Sun L Xia J Wu Q Wang Y . Gut microbiota-bile acid-interleukin-22 axis orchestrates polycystic ovary syndrome. Nat Med. (2019) 25:1225–33. doi: 10.1038/s41591-019-0509-0, PMID: 31332392 PMC7376369

[56] Fan L Qi Y Qu S Chen X Li A Hendi M . B. adolescentis ameliorates chronic colitis by regulating Treg/Th2 response and gut microbiota remodeling. Gut Microbes. (2021) 13:1–17. doi: 10.1080/19490976.2020.1826746, PMID: 33557671 PMC7889144

[57] Xu W Li J Wu Y Zhou J Zhong J Lv Q . CD127 Expression in Naive and Memory T Cells in HIV Patients Who Have Undergone Long-Term HAART. Lab Med. (2017) 48:57–64. doi: 10.1093/labmed/lmw053, PMID: 27760802

[58] Singh SB Carroll-Portillo A Lin HC . Desulfovibrio in the Gut: The Enemy within? Microorganisms. (2023) 11. doi: 10.3390/microorganisms11071772, PMID: 37512944 PMC10383351

[59] Matsuoka T Motozono C Hattori A Kakeya H Yamasaki S Oishi S . The effects of 5-OP-RU stereochemistry on its stability and MAIT-MR1 axis. Chembiochem. (2021) 22:672–8. doi: 10.1002/cbic.202000466, PMID: 33034934

[60] Mabire M Hegde P Hammoutene A Wan J Caer C Sayegh RA . MAIT cell inhibition promotes liver fibrosis regression via macrophage phenotype reprogramming. Nat Commun. (2023) 14:1830. doi: 10.1038/s41467-023-37453-5, PMID: 37005415 PMC10067815

[61] Fenwick C Joo V Jacquier P Noto A Banga R Perreau M . T-cell exhaustion in HIV infection. Immunol Rev. (2019) 292:149–63. doi: 10.1111/imr.12823, PMID: 31883174 PMC7003858

[62] Lorvik KB Meyer-Myklestad MH Kushekar K Handeland C Medhus AW Lund-Iversen M . Enhanced Gut-Homing Dynamics and Pronounced Exhaustion of Mucosal and Blood CD4(+) T Cells in HIV-Infected Immunological Non-Responders. Front Immunol. (2021) 12:744155. doi: 10.3389/fimmu.2021.744155, PMID: 34691047 PMC8529151

[63] Gherardin NA Souter MN Koay HF Mangas KM Seemann T Stinear TP . Human blood MAIT cell subsets defined using MR1 tetramers. Immunol Cell Biol. (2018) 96:507–25. doi: 10.1111/imcb.12021, PMID: 29437263 PMC6446826

[64] Bernink JH Peters CP Munneke M te Velde AA Meijer SL Weijer K . Human type 1 innate lymphoid cells accumulate in inflamed mucosal tissues. Nat Immunol. (2013) 14:221–9. doi: 10.1038/ni.2534, PMID: 23334791

[65] O'Connor JB Fouquier J Neff CP Sterrett JD Marden T Fiorillo S . Agrarian Diet Improves Metabolic Health in HIV-positive Men with Prevotella-Rich Microbiomes: Results from a Randomized Trial. Res Sq. (2024). doi: 10.21203/rs.3.rs-5349309/v1, PMID: 41294355 PMC12710365

[66] Estes J Baker JV Brenchley JM Khoruts A Barthold JL Bantle A . Collagen deposition limits immune reconstitution in the gut. J Infect Dis. (2008) 198:456–64. doi: 10.1086/590112, PMID: 18598193 PMC2683984

[67] Kantele A Zivny J Häkkinen M Elson CO Mestecky J . Differential homing commitments of antigen-specific T cells after oral or parenteral immunization in humans. J Immunol. (1999) 162:5173–7. 10227989

[68] Masopust D Jiang J Shen H Lefrançois L . Direct Analysis of the Dynamics of the Intestinal Mucosa CD8 T Cell Response to Systemic Virus Infection. J Immunol. (2001) 166:2348–56. doi: 10.4049/jimmunol.166.4.2348, PMID: 11160292

[69] Tang VA Rosenthal KL . Intravaginal infection with herpes simplex virus type-2 (HSV-2) generates a functional effector memory T cell population that persists in the murine genital tract. J Reprod Immunol. (2010) 87:39–44. doi: 10.1016/j.jri.2010.06.155, PMID: 20688399

[70] O'Neil TR Hu K Truong NR Arshad S Shacklett BL Cunningham AL . The Role of Tissue Resident Memory CD4 T Cells in Herpes Simplex Viral and HIV Infection. Viruses. (2021) 13. doi: 10.3390/v13030359, PMID: 33668777 PMC7996247

[71] Kiniry BE Li S Ganesh A Hunt PW Somsouk M Skinner PJ . Detection of HIV-1-specific gastrointestinal tissue resident CD8(+) T-cells in chronic infection. Mucosal Immunol. (2018) 11:909–20. doi: 10.1038/mi.2017.96, PMID: 29139476 PMC5953759

[72] Moylan DC Goepfert PA Kempf MC Saag MS Richter HE Mestecky J . Diminished CD103 (αEβ7) Expression on Resident T Cells from the Female Genital Tract of HIV-Positive Women. Pathog Immun. (2016) 1:371–87. doi: 10.20411/pai.v1i2.166, PMID: 28164171 PMC5288734

[73] Laidlaw BJ Zhang N Marshall HD Staron MM Guan T Hu Y . CD4+ T cell help guides formation of CD103+ lung-resident memory CD8+ T cells during influenza viral infection. Immunity. (2014) 41:633–45. doi: 10.1016/j.immuni.2014.09.007, PMID: 25308332 PMC4324721

[74] Roosenboom B Wahab PJ Smids C Groenen MJM van Koolwijk E van Lochem EG . Intestinal CD103+CD4+ and CD103+CD8+ T-Cell Subsets in the Gut of Inflammatory Bowel Disease Patients at Diagnosis and During Follow-up. Inflammatory Bowel Dis. (2019) 25:1497–509. doi: 10.1093/ibd/izz049, PMID: 30918941 PMC6701511

[75] Zhang Z Cheng L Zhao J Li G Zhang L Chen W . Plasmacytoid dendritic cells promote HIV-1-induced group 3 innate lymphoid cell depletion. J Clin Invest. (2015) 125:3692–703. doi: 10.1172/JCI82124, PMID: 26301812 PMC4588300

[76] Kloverpris HN Kazer SW Mjosberg J Mabuka JM Wellmann A Ndhlovu Z . Innate Lymphoid Cells Are Depleted Irreversibly during Acute HIV-1 Infection in the Absence of Viral Suppression. Immunity. (2016) 44:391–405. doi: 10.1016/j.immuni.2016.01.006, PMID: 26850658 PMC6836297

[77] Khoury G Rajasuriar R Cameron PU Lewin SR . The role of naive T-cells in HIV-1 pathogenesis: an emerging key player. Clin Immunol. (2011) 141:253–67. doi: 10.1016/j.clim.2011.09.002, PMID: 21996455

[78] Spaan M Hullegie SJ Beudeker BJ Kreefft K van Oord GW Groothuismink ZM . Frequencies of Circulating MAIT Cells Are Diminished in Chronic HCV, HIV and HCV/HIV Co-Infection and Do Not Recover during Therapy. PloS One. (2016) 11:e0159243. doi: 10.1371/journal.pone.0159243, PMID: 27416100 PMC4945024

[79] Cosgrove C Ussher JE Rauch A Gartner K Kurioka A Huhn MH . Early and nonreversible decrease of CD161++ /MAIT cells in HIV infection. Blood. (2013) 121:951–61. doi: 10.1182/blood-2012-06-436436, PMID: 23255555 PMC3567342

[80] Khaitan A Kilberg M Kravietz A Ilmet T Tastan C Mwamzuka M . HIV-Infected Children Have Lower Frequencies of CD8+ Mucosal-Associated Invariant T (MAIT) Cells that Correlate with Innate, Th17 and Th22 Cell Subsets. PloS One. (2016) 11:e0161786. doi: 10.1371/journal.pone.0161786, PMID: 27560150 PMC4999196

[81] Vinton C Wu F Rossjohn J Matsuda K McCluskey J Hirsch V . Mucosa-Associated Invariant T Cells Are Systemically Depleted in Simian Immunodeficiency Virus-Infected Rhesus Macaques. J Virol. (2016) 90:4520–9. doi: 10.1128/JVI.02876-15, PMID: 26912615 PMC4836342

[82] Dias J Sobkowiak MJ Sandberg JK Leeansyah E . Human MAIT-cell responses to Escherichia coli: activation, cytokine production, proliferation, and cytotoxicity. J Leukoc Biol. (2016) 100:233–40. doi: 10.1189/jlb.4TA0815-391RR, PMID: 27034405 PMC4946616

[83] Mullins TLK Li SX Bethel J Goodenow MM Hudey S Sleasman JW . Sexually transmitted infections and immune activation among HIV-infected but virally suppressed youth on antiretroviral therapy. J Clin Virol. (2018) 102:7–11. doi: 10.1016/j.jcv.2018.02.001, PMID: 29454196 PMC5889960

[84] Vital M Karch A Pieper DH . Colonic Butyrate-Producing Communities in Humans: an Overview Using Omics Data. mSystems. (2017) 2. doi: 10.1128/mSystems.00130-17, PMID: 29238752 PMC5715108

[85] Rubinchik S Seddon A Karlyshev AV . Molecular mechanisms and biological role of Campylobacter jejuni attachment to host cells. Eur J Microbiol Immunol (Bp). (2012) 2:32–40. doi: 10.1556/EuJMI.2.2012.1.6, PMID: 24611119 PMC3933988

[86] Siddiq M O'Flanagan H Richardson D Llewellyn CD . Factors associated with sexually transmitted shigella in men who have sex with men: a systematic review. Sex Transm Infect. (2023) 99:58–63. doi: 10.1136/sextrans-2022-055583, PMID: 36283806

[87] Louis P Young P Holtrop G Flint HJ . Diversity of human colonic butyrate-producing bacteria revealed by analysis of the butyryl-CoA:acetate CoA-transferase gene. Environ Microbiol. (2010) 12:304–14. doi: 10.1111/j.1462-2920.2009.02066.x, PMID: 19807780

[88] Plassart C Mauvais F Heurte J Sautereau J Legeay C Bouvet P . First case of intra-abdominal infection with Clostridium disporicum. Anaerobe. (2013) 19:77–8. doi: 10.1016/j.anaerobe.2012.12.002, PMID: 23247006

[89] Hiippala K Barreto G Burrello C Diaz-Basabe A Suutarinen M Kainulainen V . Novel Odoribacter splanchnicus Strain and Its Outer Membrane Vesicles Exert Immunoregulatory Effects in vitro. Front Microbiol. (2020) 11:575455. doi: 10.3389/fmicb.2020.575455, PMID: 33281770 PMC7689251

[90] Wan X Wang S Wang M Liu J Zhang Y . Identification of Peptoniphilus harei From Blood Cultures in an Infected Aortic Aneurysm Patient: Case Report and Review Published Literature. Front Cell Infect Microbiol. (2021) 11:755225. doi: 10.3389/fcimb.2021.755225, PMID: 35004343 PMC8730293

[91] Serafini N Klein Wolterink RG Satoh-Takayama N Xu W Vosshenrich CA Hendriks RW . Gata3 drives development of RORgammat+ group 3 innate lymphoid cells. J Exp Med. (2014) 211:199–208. doi: 10.1084/jem.20131038, PMID: 24419270 PMC3920560

[92] Jabeen MF Hinks TSC . MAIT cells and the microbiome. Front Immunol. (2023) 14:1127588. doi: 10.3389/fimmu.2023.1127588, PMID: 36911683 PMC9995591

[93] Hammer SM Sobieszczyk ME Janes H Karuna ST Mulligan MJ Grove D . Efficacy trial of a DNA/rAd5 HIV-1 preventive vaccine. N Engl J Med. (2013) 369:2083–92. doi: 10.1056/NEJMoa1310566, PMID: 24099601 PMC4030634

[94] Neff CP Chain JL MaWhinney S Martin AK Linderman DJ Flores SC . Lymphocytic alveolitis is associated with the accumulation of functionally impaired HIV-specific T cells in the lung of antiretroviral therapy-naive subjects. Am J Respir Crit Care Med. (2015) 191:464–73. doi: 10.1164/rccm.201408-1521OC, PMID: 25536276 PMC4351596

[95] Thompson LR Sanders JG McDonald D Amir A Ladau J . A communal catalogue reveals Earth’s multiscale microbial diversity. Nature. (2017) 551:457–63. doi: 10.1038/nature24621, PMID: 29088705 PMC6192678

[96] McDonald D Jiang Y Balaban M Cantrell K Zhu Q Gonzalez A . Greengenes2 unifies microbial data in a single reference tree. Nat Biotechnol. (2024) 42:715–8. doi: 10.1038/s41587-023-01845-1, PMID: 37500913 PMC10818020

[97] McDonald D Price MN Goodrich J Nawrocki EP DeSantis TZ Probst A . An improved Greengenes taxonomy with explicit ranks for ecological and evolutionary analyses of bacteria and archaea. ISME J. (2012) 6:610–8. doi: 10.1038/ismej.2011.139, PMID: 22134646 PMC3280142

[98] Bolyen E Rideout JR Dillon MR Bokulich NA Abnet CC Al-Ghalith GA . Reproducible, interactive, scalable and extensible microbiome data science using QIIME 2. Nat Biotechnol. (2019) 37:852–7. doi: 10.1038/s41587-019-0209-9, PMID: 31341288 PMC7015180

[99] Janssen S McDonald D Gonzalez A Navas-Molina JA Jiang L Xu ZZ . Phylogenetic Placement of Exact Amplicon Sequences Improves Associations with Clinical Information. mSystems. (2018) 3. doi: 10.1128/mSystems.00021-18, PMID: 29719869 PMC5904434

[100] Zhang Z Schwartz S Wagner L Miller W . A greedy algorithm for aligning DNA sequences. J Comput Biol. (2000) 7:203–14. doi: 10.1089/10665270050081478, PMID: 10890397

[101] Anderson MJ . A new method for non-parametric multivariate analysis of variance. Austral Ecol. (2001) 26:32–46.

[102] Sayers EW Bolton EE Brister JR Canese K Chan J Comeau DC . Database resources of the national center for biotechnology information. Nucleic Acids Res. (2022) 50:D20–6. doi: 10.1093/nar/gkab1112, PMID: 34850941 PMC8728269

