# Supplementary material for: Gut microbiome composition influences immunologic alterations in the blood and gut of HIV-positive and HIV-negative men who have sex with men
Source: Front Immunol. 2026 Jan 2;16:1707736. doi: 10.3389/fimmu.2025.1707736 (PMC12808405; doi:10.3389/fimmu.2025.1707736)
Supplement: Supplementary file 2 [file Table2.docx]

**Supplemental Table 1. CyTOF Antibody Staining Panel**

**
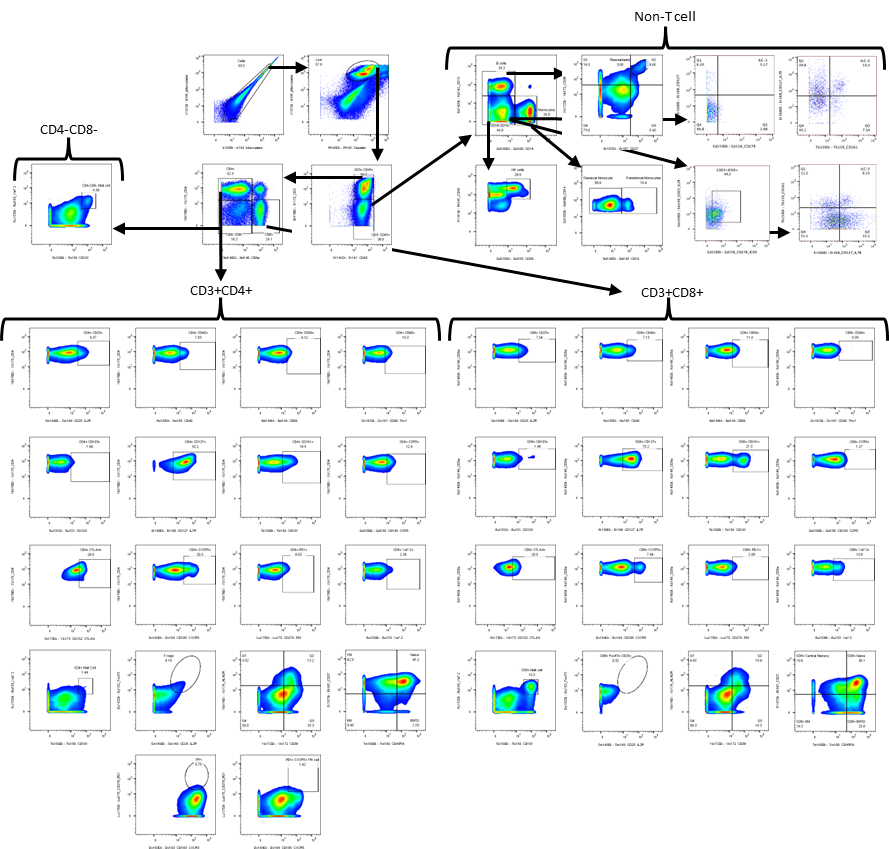
**

**Supplemental Figure 1. Representative CyTOF Gating Strategy.** Representative gating strategy of blood immune populations identified by CyTOF.

**Supplemental Table 2. Immune populations in blood and colonic biopsy**

Immune population based on gating hierarchy and a short name used in the text and figures are provided. For each immune population and tissue the number of samples (N) and the number of samples by cohort (N by cohort, with A, B, and C corresponding to HIV^-^ non-MSM, HIV^-^ MSM, and HIV+ MSM respectively), Mean by cohort and SSE (square of standard error) by cohort are provided to support effect size calculations in future studies. pF and pFAdjFDR are the p-value on the F statistic on the linear model (analyte ~ cohort), and the corresponding FDR-adjusted statistic. sAB, sAC, and sBC are significance codes (s=significant, p < 0.05, Tukey HSD; ns=not significant) on the pairwise comparisons of means with A, B, and C representing cohorts as defined above. Results for age-adjusted analysis are also shown (analyte ~ age + cohort), with aa.pPartialF representing the p-value of the partial F statistic, and aa.pPartialFAdjFDR representing the corresponding FDR-adjusted value. Significance codes are also shown for pairwise comparisons of means from the age adjusted models (aa.sAB, aa.sAC, and aa.sBC), as described above.


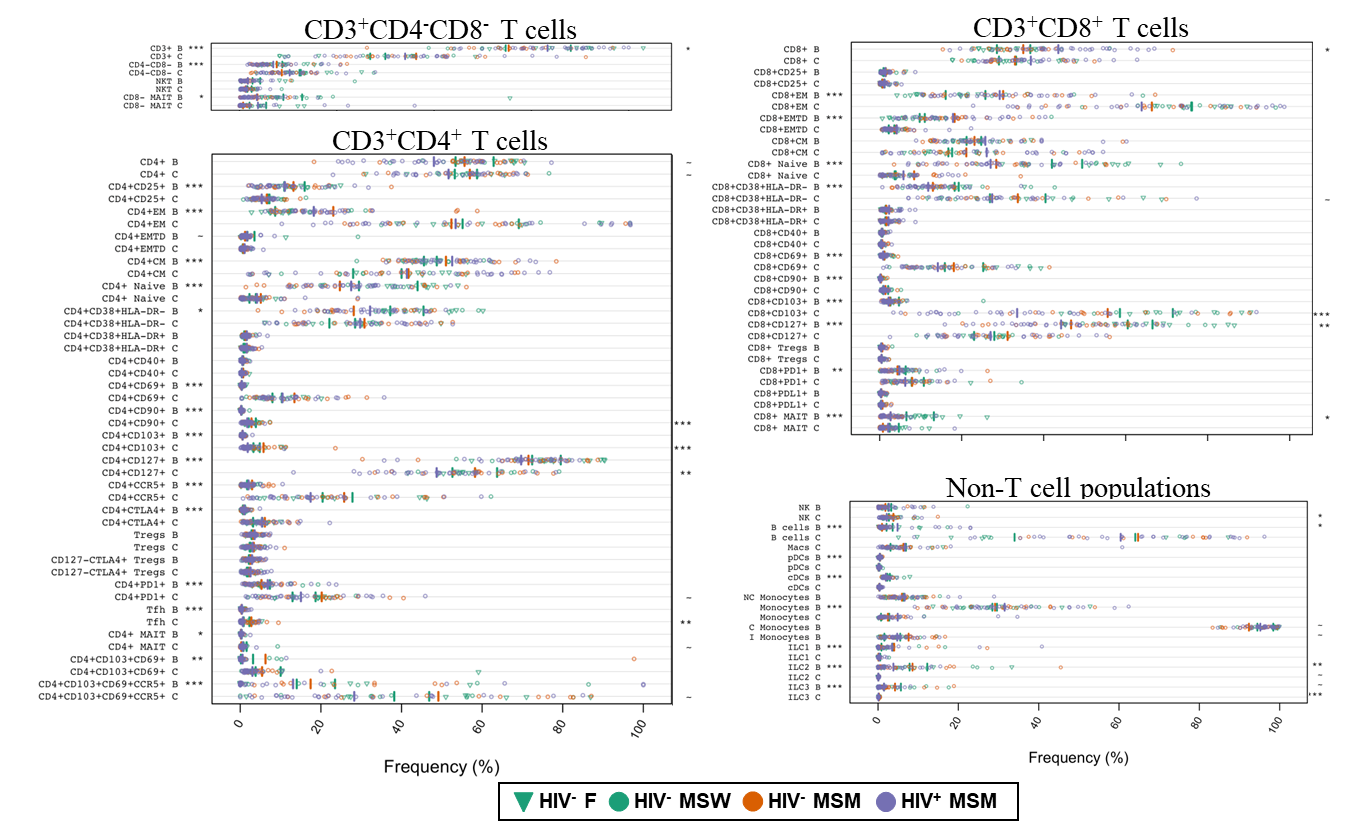


**Supplemental Figure 2. Statistically significant difference in blood and colonic immune cell populations across cohorts.** Each point, colored by cohort, represents one participant. Colored vertical bars within each row of data indicate mean by cohort. Values for both blood (B) and colonic biopsy (C) are shown. On the left side of the plot, significant differences in mean value between blood (B) and colonic biopsy (C) are indicated by asterisks (*)(paired t-test, FDR adjusted). On the right side of the plot, significant differences across cohorts (unadjusted partial F statistic from linear model of frequency ~ cohort after adjusting for age, day, and gender. Pairwise cross-cohort comparisons adjusted with Tukey HSD) are indicated by asterisks (*). Significance codes are defined as ‘***’ [0, 0.001], ‘**’ (0.001, 0.01], ‘*’ (0.01, 0.05); with square brackets indicating endpoints included in the interval.


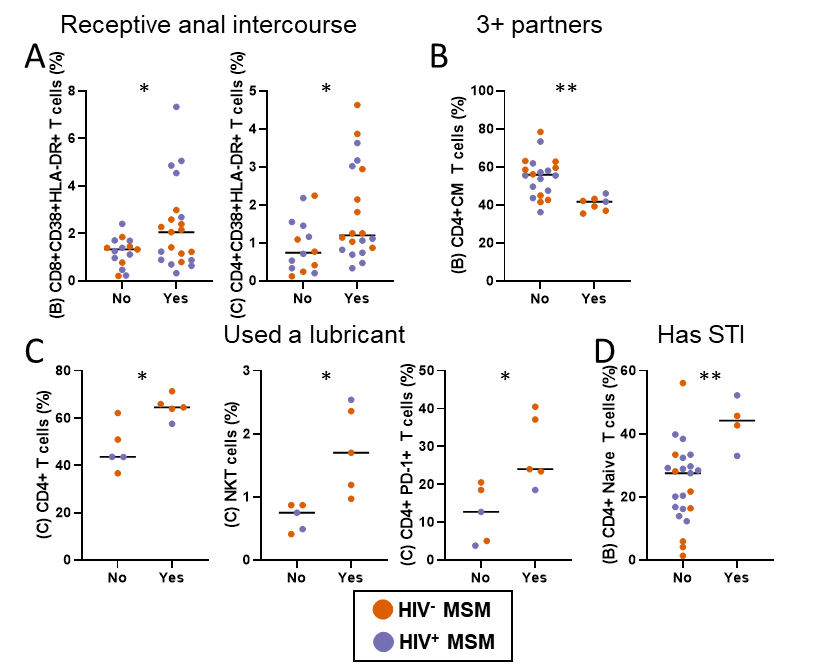


**Supplemental Figure 3. Sexual behaviors, Lubricants and sexually transmitted infections are associated with differences in immune populations.**  **(A)** Increased levels of colonic and blood activated T cells are associated with Receptive anal intercourse (RAI), after adjusting for HIV status. Solid lines represent mean values. No=have not participated in RAI; Yes=have participated in RAI. (* < 0.05). **(B)** Having 3 or more partners within the 6 months of the study date is associated with a decrease in central memory (CM) CD4+ T cells (** < 0.01) **(C)** Total CD4+ T cells, NKT cells and CD4+ PD-1+ T cells are increased with using lubricant. No=have not used lubricants in the anal region during intercourse. (* < 0.05). **(D)** Decreased levels in blood naïve cells are associated with a new sexually transmitted infection (STI). N=no new STI infection in the 12 months prior to visit date, Yes=new STI (** < 0.01)

**Supplemental Table 3. ASV differences by cohort by tissue.** Statistical summary of differences by cohort for ASVs with FDR-adjusted p-value < 0.1 (F statistic from linear model of the form relative abundance ~ cohort + reads) in either feces or colonic biopsy. ASV: Last two levels of the taxonomic assignment of the ASV. pFadjFDR.fm, .cm: FDR-adjusted p-value for fecal microbe and colonic microbe, respectively. pABSig, pACSig, pBCSig (.fm=fecal microbe, .cm=colonic microbe): Whether or not pairwise comparisons across cohorts were significantly different (Tukey’s HSD), with A, B, and C corresponding to HIV^-^ non-MSM, HIV^-^ MSM, and HIV+ MSM respectively. SignifSummary (.fm, cm): statement indicating direction and cohort for significant differences. Comparison is relative to the HIV- non-MSM control unless otherwise specified. Fecal v. Colonic: Comparison across tissues. NA = not applicable (ASV not observed in this tissue), NS=not significant.

**
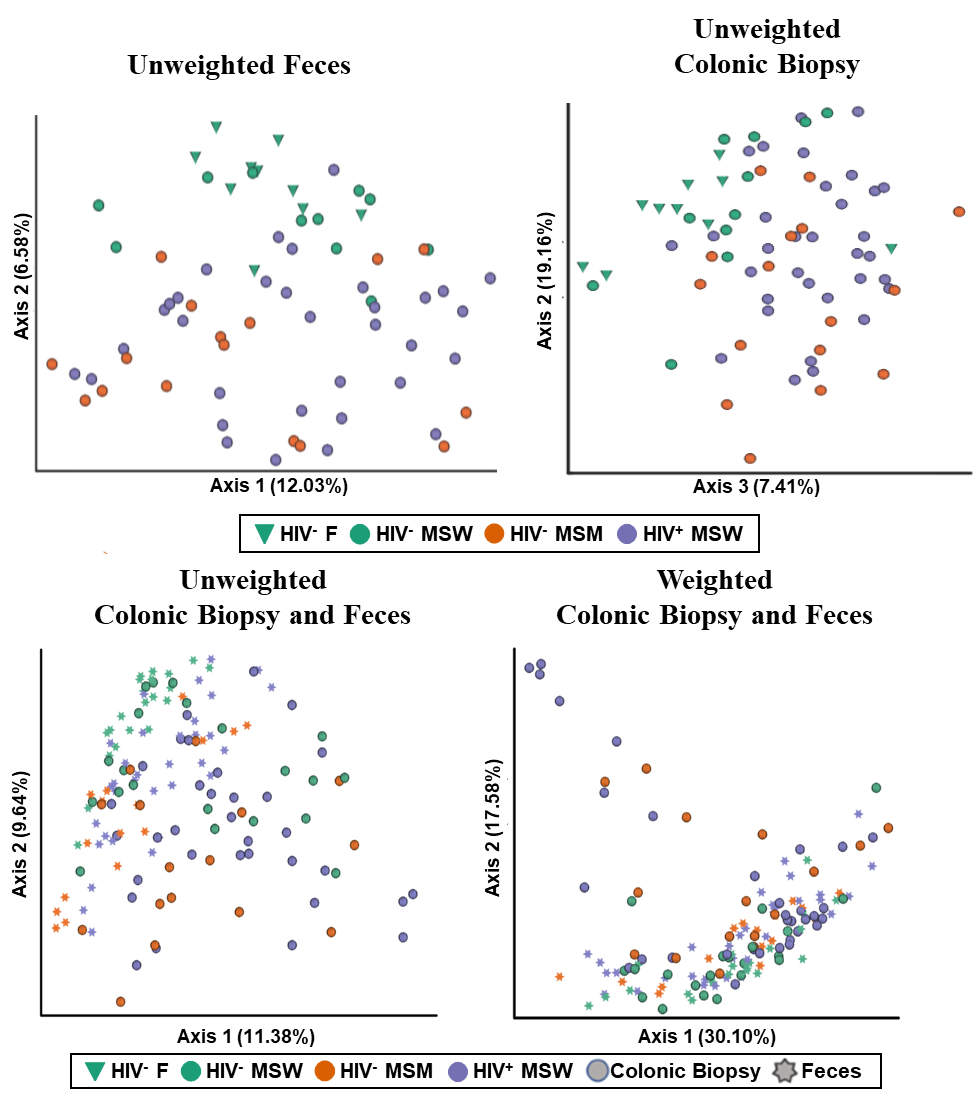
Supplemental Figure 4. PCoA of ASVs in Feces and Colonic Biopsy**. PCoA Clustering of Canberra distance matrices of participants based on immune data from colonic biopsy and blood. Points are colored by cohort with green: HIV- not MSM, orange: HIV- MSM and purple: HIV+ MSM. Vector magnitude of immune populations are related to importance, calculated across all dimensions using QIIME 2.


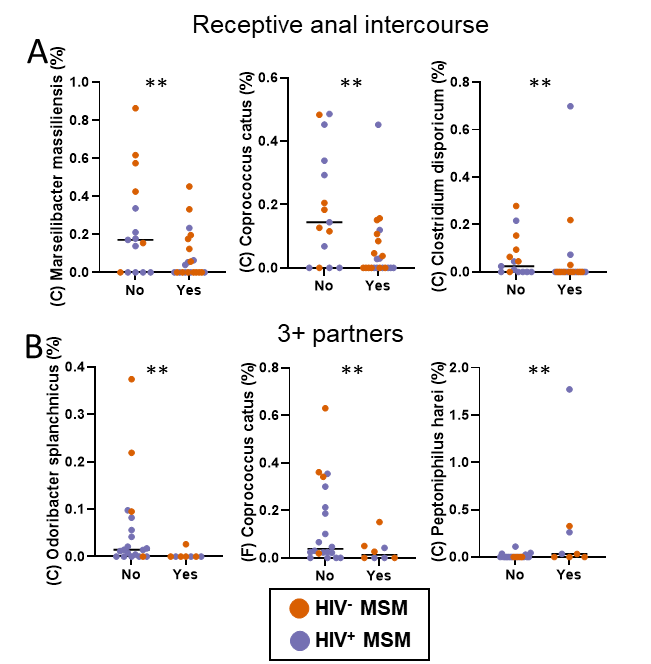


**Supplemental Figure 5. Sexual behaviors are associated with differences in immune populations.**  **(A)** Reduced relative abundance of bacteria taxa is associated with Receptive anal intercourse (RAI). Solid lines represent mean values. No=have not participated in RAI; Yes=have participated in RAI. (** < 0.01) **(B)** Having 3 or more partners within the 6 months of the study date is associated with changes in relative abundance or bacteria taxa (** < 0.01)**.**

**Supplemental Table 4. Network Edges**. Detailed description of all network edges. Assays: Assays or sample types for Analyte 1 and Analyte 2 respectively (bc=blood cells, cc=colonic cells, fm=fecal microbe, cm=colonic microbe). Analyte 1: immune cell population on y axis. Analyte 2: immune cell population or microbe on x axis. BLAST name: Scientific name returned by BLAST search on FASTA sequence, coupled with percent identity (%ID). # indicates multiple BLAST results at same level of %ID. For sequences with > 97% identity, the scientific name was used for Analyte 2. Otherwise, the taxa assigned by QIIME was used. n: number of samples included in the regression model. nNot0Ana2: number of non-zero values for Analyte 2. adjRsq: Adjusted R^2^ value for fitted model. pF: p-value from F statistic of model. pSHIV- non-MSM, pSHIV-MSM, pHIV+MSM: p-values for slope different than 0 for each cohort. Slope pattern: 1 term for each of HIV^-^ non-MSM, HIV^-^ MSM, and HIV+ MSM respectively. Slopes that were significantly different than 0 were encoded as p = positive slope and n = negative slope, with ~ = not significantly different than 0

**
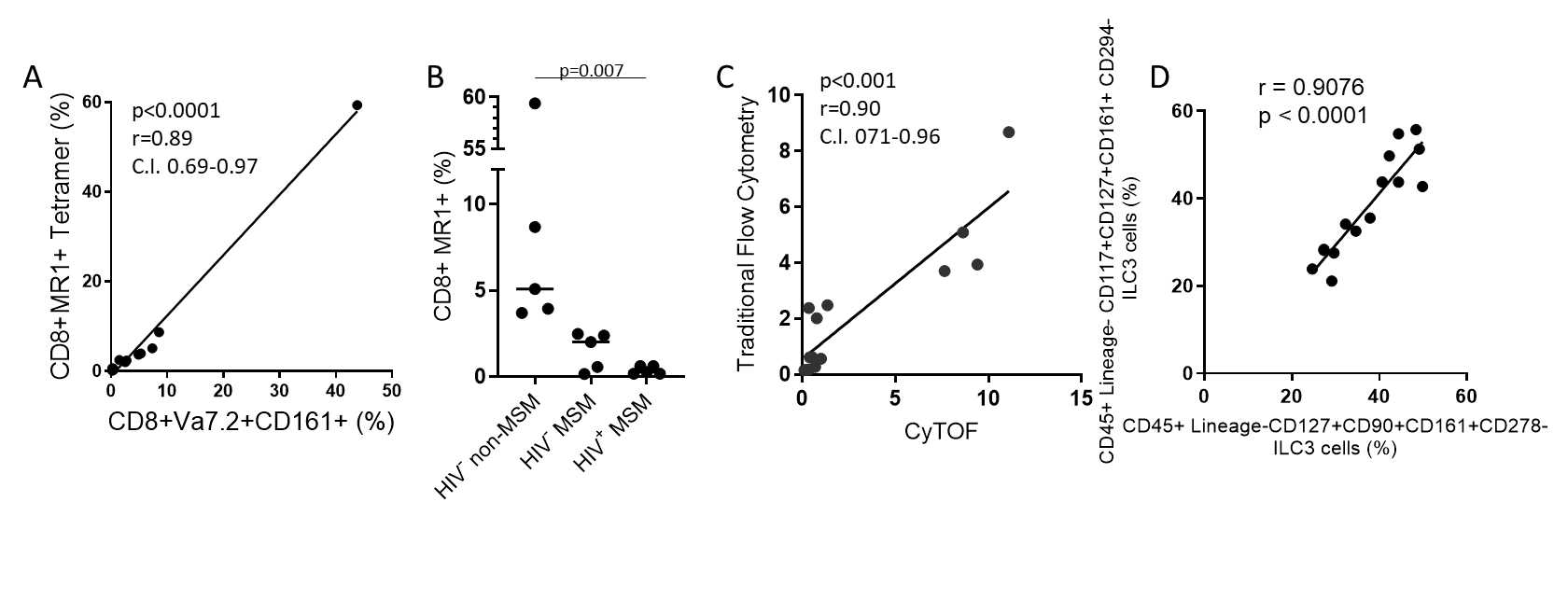
Supplemental Figure 6**. **Blood MAIT cells and colonic ILC3 identification and quantification confirmed by traditional flow cytometry.** **(A)** Correlation of blood MAIT cells stained for CD8+Va7.2+CD161+ or a MR1 tetramer. **(B)** Blood MAIT cell frequencies were determined on a subset of subjects from each cohort. **(C)** Correlation of frequency of Blood MAIT cells found with traditional flow cytometer or CyTOF. **(D)** Correlation of frequency of colonic ILC3s identified by being CD45⁺Lin⁻CD127⁺CD161⁺CD90⁺ICOS^-^ or CD45⁺Lin⁻CD117^+^CD127⁺CD161⁺CRTH2^-^. Statistical significance was calculated using Kruskal-Wallis or Pearson correlation coefficient. Significance codes are defined as ‘***’ [0, 0.001], ‘**’ (0.001, 0.01], ‘*’ (0.01, 0.05); with square brackets indicating endpoints included in the interval.
